# Pattern and Epidemiology of Poisoning in the East African Region: A Literature Review

**DOI:** 10.1155/2016/8789624

**Published:** 2016-11-01

**Authors:** Dexter Tagwireyi, Patience Chingombe, Star Khoza, Mandy Maredza

**Affiliations:** ^1^Drug and Toxicology Information Service (DaTIS), School of Pharmacy, College of Health Sciences, University of Zimbabwe, P.O. Box A178, Avondale, Harare, Zimbabwe; ^2^Department of Clinical Pharmacology, College of Health Sciences, University of Zimbabwe, P.O. Box A178, Avondale, Harare, Zimbabwe; ^3^School of Public Health, Faculty of Health Sciences, University of Witwatersrand, Johannesburg, South Africa

## Abstract

The establishment and strengthening of poisons centres was identified as a regional priority at the first African regional meeting on the Strategic Approach to International Chemicals Management (SAICM) in June 2006. At this meeting, the possibility of a subregional poisons centre, that is, a centre in one country serving multiple countries, was suggested. The WHO Headquarters following consultation with counterparts at the WHO Regional Office for Africa (AFRO) and the SAICM Africa Regional Focal Point successfully submitted a proposal to the SAICM Quick Start Programme (QSP) Trust Fund Committee for a feasibility study into a subregional poisons centre in the Eastern Africa subregion. However, before such a study could be conducted it was deemed necessary to carry out a literature review on the patterns and epidemiology of poisoning in this region so as to inform the feasibility study. The current paper presents the results of this literature review. The literature search was done in the months of June and July 2012 by two independent reviewers with no language or publication date restrictions using defined search terms on PUBMED. After screening, the eventual selection of articles for review and inclusion in this study was done by a third reviewer.

## 1. Background

The establishment and strengthening of poisons centres was identified as a regional priority at the first African regional meeting on the Strategic Approach to International Chemicals Management (SAICM) in June 2006. At this meeting, the possibility of a subregional poisons centre, that is, a centre in one country serving multiple countries, was suggested. However, at its fifth meeting in January 2010, the SAICM Africa Core Group, which comprises representatives from all subregions, noted a continuing lack of progress on this issue and requested that proposals be developed to address this. The World Health Organization (WHO) has had a long-standing programme of work directed at assisting countries to establish and strengthen poisons centres. As such, WHO Headquarters following consultation with counterparts at the WHO Regional Office for Africa (AFRO) and the SAICM Africa Regional Focal Point successfully submitted a proposal to the SAICM Quick Start Programme (QSP) Trust Fund Committee for a feasibility study into a subregional poisons centre in the Eastern Africa subregion. The results of this feasibility study have since been published elsewhere [1]. In addition, a summary of the project has also been recently published [2]. However, before the feasibility study could be done and in order to get a better understanding of the problem of poisoning in this region, a systematic literature review on the patterns and epidemiology of poisoning in the Eastern African subregion (as defined by UNOSTAT) was carried out. This paper presents the results of this work. A recent impact review of the SAICM QSP programme mentioned the literature review on poisoning in Africa as one of the useful outputs of the QSP projects; however it was also stated in that same review that although a lot of useful information was gathered by the projects, this information is not readily available through the SAICM Secretariat. As a result of the above and the perceived usefulness of the data gathered during this review, it was decided to publish this work in an open access journal. It is hoped that this literature review will provide a valuable source for clinicians, toxicologists, and researchers alike who may wish to acquire a quick overview of issues relating to poisoning in the East African region. In addition, this paper is aimed at making literature on poisoning in Africa more readily accessible to the scientific and health communities, especially given the fact that toxicovigilance systems in Africa are still nascent [3] and thus such systematic data is hardly available.

The countries covered in the review were Burundi, Comoros, Djibouti, Eritrea, Ethiopia, Kenya, Madagascar, Malawi, Mauritius, Mozambique, Rwanda, Seychelles, Tanzania, Uganda, Zambia, and Zimbabwe.

## 2. Methods

In order to describe the patterns and epidemiology of poisoning in the subregion, a literature search was conducted. The literature search was done in the months of June and July 2012 by two independent reviewers with no language or publication date restrictions. For the literature search, MEDLINE (via PubMed) was searched using the following search strategy: (epidemiology OR incidence OR prevalence OR patterns) AND (poisoning OR snakebite OR scorpion sting OR pesticide OR organophosphate poisoning OR envenomation OR toxicity OR intoxication) AND africa OR (burundi OR comoros OR djibouti OR eritrea OR ethiopia OR kenya OR madagascar OR malawi OR mauritius OR mozambique OR rwanda OR seychelles OR tanzania OR uganda OR zambia OR zimbabwe). The same search strategy was used on Google Scholar. Where no scholarly articles were identified for a country, a further literature search using “poisoning” and the name of the country was done on https://www.google.co.zw/.

Potential articles for inclusion were identified after screening titles and abstracts. The final articles were included in the review after reading the full articles. Relevant references from these articles that were not identified via the bibliographic searches were sought for and the articles analysed. From the identified articles, epidemiological parameters such as case fatality rates, mortality rates, prevalence, and incidence were identified and reported. In addition, National Health Profiles from the study countries were evaluated to identify statistics on poisoning incidences or related parameters. Members of a project Steering Group largely composed of poisons centre specialists from Ghana, South Africa, Kenya, and Zimbabwe, as well as the UK and the World Health Organization, were also requested to suggest any grey literature that they may be aware of and the investigators also provided a list of literature that they were aware of. Case reports were also included in the review. The eventual selection of articles for review and inclusion was done by a third reviewer.

## 3. Results

Table 1 shows the characteristics of the studies included in this review. Although the main aim of the study was to describe epidemiological parameters, poisoning case reports are mentioned for the different countries. The results are presented according to countries.

### 3.1. Burundi

WHO has estimated that there were 7.8 deaths per 100,000 persons due to unintentional poisoning in 2004 [4]. No published studies of the epidemiology of poisoning in Burundi were found.

### 3.2. Comoros

WHO has estimated that there were 1.7 deaths per 100,000 persons due to unintentional poisoning in 2004 [4]. No published studies of the epidemiology of poisoning in Comoros were found.

### 3.3. Djibouti

WHO has estimated that there were 3.9 deaths per 100,000 people due to unintentional poisoning in 2004 [4]. Literature on the patterns and epidemiology of poisoning in Djibouti were scanty. There was a prospective descriptive study of childhood acute accidental poisoning with kerosene in Djibouti done by Benois and colleagues [5]. Of the 17 cases involved, 35% were asymptomatic and were discharged with 41% developing pneumonia. Seignot and others [6] also reported on a fatal envenomation in a 44-year-old male who was bitten by an African viper* (Echis carinatus)* in Djibouti. Larréché et al. [7] conducted a retrospective study of snakebite victims for the period from October 1994 to May 2006. In this study, the authors compared the normalisation of the haemostasis disorders with early administration of antivenin versus delayed administration. Aigle and colleagues [8] also described a prospective study of stingray stings between July 2008 and July 2009. A total of twelve stings were treated during the study period. There were however no specific studies describing the epidemiology of poisoning in general in the country.

### 3.4. Eritrea

WHO has estimated that there were 3.7 deaths per 100,000 persons due to unintentional poisoning in 2004 [4]. No published studies of the epidemiology of poisoning in Eritrea were found.

### 3.5. Ethiopia

WHO has estimated that there were 3.5 deaths per 100,000 persons due to unintentional poisoning in 2004 [4]. A number of papers have been published concerning poisoning in Ethiopia. Aseffa and colleagues [10] reported on an outbreak of food poisoning resulting from* Salmonella Newport*. This occurred between December 31, 1991, and January 4, 1992, and involved students at a Medical College in the country. Out of 344 students, 79 (23%) had clinical symptoms of food poisoning from the bacteria. The main symptoms of the food poisoning episode were malaise, diarrhoea, and abdominal cramps. There were no fatalities in this outbreak. Aga and Geyid [18] also reported on an outbreak of food poisoning resulting from eating food contaminated with* Datura stramonium* during the period of July and August 1984. In their paper, the authors reported on 688 cases seen at the study clinics exhibiting unusual signs and symptoms, with 33 requiring hospitalisation for intensive medical care. Nine deaths were reported (case fatality rate of 1.31%). Poisoning was identified as the second most common method of attempted suicide in a district of Ethiopia, accounting for 42.4% of the 332 lifetime suicide attempt respondents [11] from a community based survey which covered a total of 5259 houses with 12 531 residents above 15 years of age. Poisoning was second only to hanging which was reported in 48% of the suicide attempts. The authors reported that poisoning was used more commonly by women than by men with strong detergents and rodenticides being the most frequently used poisons.

Poisoning from organophosphates was also identified as an important cause of poisoning from studies in Ethiopia. Abebe [9] reported a high case fatality rate of 20% resulting from organophosphate poisoning in 50 Ethiopian patients. In a prospective study reported by Selassie [16], a total of 23 cases of organophosphate poisoning were admitted during the period from January 1996 to January 1997. The male to female ratio was reported as 1 : 2.83 with the most common clinical findings being vomiting and abdominal pains. There were no deaths reported in this study. A study by Melaku and colleagues [13] reported that organophosphate poisoning accounted for 4.7% (168/3548) of medical intensive care unit (MICU) admissions over a 15-year period (1985 to 2000) at Tikur Anbessa Specialised Teaching Hospital in Ethiopia [13]. Based on results of a retrospective study of acute poisoning admissions to Gondar University Teaching Hospital, Abula and Wondmikun [12] reported that organophosphate poisoning comprised 41.5% of all acutely poisoned patients with a case fatality rate of 2.4%. Desalew and colleagues [14] also identified organophosphates as an important cause of poisoning in adults in their study where they reviewed medical case files of 116 acutely poisoned adult patients (greater than 13 years) presenting to Tikur Anbessa Specialised University Hospital for the period from January 2007 to December 2008. From the results of their study, most (96.5%) of the cases were intentional self-harm cases with household cleaning agents being the leading toxicants used (43.1%) followed by organophosphates (21.6%) and phenobarbitone (10.3%). The case fatality rate from this study was reported to be 8.6% with most deaths occurring from organophosphate (5) and phenobarbitone (3) ingestion. Most patients stayed only one day in hospital (76%). The only study that reported on incidence of poisoning in Ethiopia was a risk assessment of staphylococcal poisoning due to consumption of informally marketed milk and home-made yoghurt in Debre Zeyit, Ethiopia [17]. Based on a mathematical model, the authors estimated that the annual incidence rate of staphylococcal poisoning in the area was 20 (90% CI: 13.9–26.9) per 1000 people.

### 3.6. Kenya

WHO has estimated that there were 3.4 deaths per 100,000 people due to unintentional poisoning in 2004 [4]. Literature from Kenya shows the existence of case reports on a wide range of poisonings including mushroom poisoning [19], snakebite from spitting cobra [20, 22], and salicylate poisoning [31]. Moreover, there are a couple of papers that have given epidemiological data on poisoning outbreaks in the country. Smith and colleagues [23] reported on an outbreak of botulism in Kenyan nomads. The authors reported that 300 Gabra were involved in the outbreak. The outbreak is said to have begun with a young adult female who prepared some sour milk traditionally in a gourd using camel milk. Sixteen people attended her funeral. Of these ten (10) fell sick in four days following the funeral and five of them died. Of the six who did not fall sick, three had taken tea with fresh camel's milk and the remaining three took nothing. Investigations including laboratory analyses showed that the sour milk had been contaminated with* Clostridium botulinum* Type A. Thus the attack rate for the entire community of 300 was 3%, and 62% for the funeral attenders. Ngindu et al. [25] also reported on an outbreak of aflatoxicosis where 20 patients with hepatitis were admitted to three hospitals in Machakos district of Kenya with a very high case fatality rate of 60%. From this report, there was laboratory evidence of aflatoxicosis with maize contaminated with levels as high as 12,000 parts per billion and liver tissue necropsy giving levels of up to 89 parts per billion. More recently, an outbreak of acute hepatotoxicity was identified among people living in Kenya's eastern and central provinces in April 2004. In this particular outbreak, by July 20, 2004, the case fatality rate among the 317 cases was 39.4% (125 deaths had occurred). This was defined as one of the largest and most severe outbreaks of aflatoxicosis documented worldwide [28].

Apart from epidemiological data from outbreaks, a number of hospital based toxicoepidemiological studies have also been reported in the literature for Kenya. Mwangemi [21] reviewed the case notes of snakebite victims admitted to the Wajir District Hospital in Northeastern Province of Kenya for the period of 1974 to 1975. They identified a total of 38 patients. The case fatality rate was 2.6%, an adult male had his leg amputated, and 2 children required massive blood transfusions. The remaining 34 patients made good clinical recovery and were allowed to go home after 1-2 weeks [21].

Kahuho [24] also did a retrospective study of patients admitted with drug and other chemical poisonings in the intensive care unit of Kenyatta National Hospital between August 1972 and April 1978. During this period, there were a total of 72 poisoned patients out of 2135 admissions. Of these 28 (case fatality rate of 36.1%) died. Organophosphates accounted for the highest number of deaths, followed by unknown chemicals. There were 25 (34.7%) children aged below 5 years of whom 13 died. Most of the admissions were as a result of exposure to organophosphates (33.3%) of whom 45.9% died. Another retrospective review in the same hospital, this time looking at hospital admissions during 2002-2003, identified 463 cases of which 458 had complete case notes [35]. The largest age category was young adults (21–30 years), accounting for 38% of cases, with children under five years accounting for 23.4% of cases. Pesticide poisoning was the cause of 43% of poisoning admissions, with a predominance of organophosphates and rodenticides. In children under five, poisoning with household products accounted for 66.4% of cases, and almost all of these were due to paraffin ingestion.

A three-year retrospective review of admissions to 19 hospitals identified 1904 cases of poisonings [29]. The study focused on children, who accounted for 40%. In the under-five-year age group, paraffin, drugs, and organophosphates accounted for 41.09, 23.81, and 15.17% of poisoning cases, respectively.

A hospital based case review by Mbakaya et al. [32] identified 455 cases of poisoning due to organochlorines. This was despite the discontinued use of this pesticide in the countries where it was imported from. The authors attributed the continual use of the pesticide in Kenya to weak regulatory structures that enabled the importation and usage of pesticides no longer in use in the countries of origin.

From another hospital based retrospective case review, Lang and colleagues [30] reported a case fatality rate of 2% and an incidence of 17 per 100,000 for paraffin poisoning in children aged 0 to 13 years.

Snow et al., 1994 [26], undertook a community based retrospective survey of 4712 households on snakebite. The questionnaire was designed to cover circumstances of the bite, morbidity, sequelae, treatment, and perceptions. The data collected were for bites that had occurred in 1993. There were a total of 121 bites reported by the field staff. Of these 21 were in nonresidents of the study area and a further 22 were excluded from the analyses since further investigations revealed that they had been bitten prior to 1993. The annual rate of snakebite among the population was estimated to be 150/100,000 persons per annum. Of the 66 victims with whom in-depth interviews were done, most were male (57%) and most bites occurred in the night (55%). In addition, most of the bites (73%) were to the feet. Only 26 (39%) of the interviewees could reliably describe the snake that bit them. Only 19% of the victims were bitten by potentially venomous snakes with the puff adder being the most commonly identified venomous species. There were no deaths reported from this study for 1993.

Coombs and colleagues [27] collected primary data on the incidence and severity of and species responsible for snakebites in 4 areas of Kenya: (i) Kakamega and western Kenya, (ii) Lake Baringo and Laikipia, (iii) Kilifi and Malindi, and (iv) northern Kenya. The overall average frequency of snakebite was 13.8 per 100,000 persons per year (range 1.9–67.9). The minimum rate of snakebite mortality was 0.45/100,000/year. Thirty-four of the 50 units visited reported no knowledge of death from snakebite in the last 5 years. Possible reasons for the low estimates are discussed. Traditional treatments were common, especially the use of herbal remedies and incisions at the wound site.

Cases of methanol poisoning have become quite rampant in Kenya due to illicit alcohol production. Media reports indicate that, between 1998 and 2005, more than 250 people died due to methanol poisoning in the country. In 1998 more than 80 people died in Nairobi after drinking chang'aa (traditional brew). Similarly, hospital admissions at Kenyatta National Hospital indicate that, in 2000, 512 people were admitted for chang'aa intoxication. Of these, 137 died (case fatality rate of 27%) and 20 became blind with others becoming visually impaired and physically disabled [33]. Fifty more deaths due to methanol poisoning were reported in Machakos in 2005 [34]. In response to this emergency, local brews were legalized through the Alcoholic Drinks Control Act 2010 to ensure quality control [116].

### 3.7. Madagascar

WHO has estimated that there were 2.9 deaths per 100,000 people due to unintentional poisoning in 2004 [4]. From published literature, the pattern of poisoning in Madagascar appears to be skewed toward poisoning from natural toxins with case reports of poisoning by puffer fish [47], snakebite by* Madagascarophis (Colubrida opisthoglypha)* [37], and food-induced botulism [36]. Moreover, outbreaks after ingestion of sea food have been reported and do provide some good epidemiological data. Habermehl and others [38] reported on a single outbreak of severe ciguatera/ciguatera-like poisoning in Madagascar after ingestion of shark meat. In this outbreak, more than 500 people were poisoned of whom 98 died with a case fatality rate of 20%. Boisier and colleagues also reported on another mass poisoning after ingestion of a shark* (Carcharhinus leucas)* [43]. In this episode, a case fatality rate of 30% was reported among the 200 poisoned inhabitants of Manakara, a medium-sized town on the southeast coast of Madagascar. Similarly, Ramialiharisoa and others [40, 41] reported on a single outbreak of shark poisoning which occurred in Vohipeno, east coastal region of Madagascar, where 600 people were affected. In this outbreak, four deaths occurred and clinical symptoms of 310 cases admitted in the hospital suggested a ciguatera/ciguatera-like poisoning. The sea turtle has also resulted in mass food poisoning in the Antalaha district of Madagascar [42]. This poisoning episode which affected about 60 people had an attack rate of 48% and a case fatality rate of 7.7%. In total, between January 1993 and January 1998, 19 outbreaks of food poisonings occurred as a result of sea food consumption [44]. These outbreaks affected 1789 people of whom 102 died (case fatality rate of 6%). Consumption of shark and turtle meat accounted for 70% of cases. Ribes and coworkers [45] conducted a knowledge, attitude, and practice (KAP) survey concerning seafood poisoning. This was carried out in 560 villages with 585,000 people along the Madagascar coast. From the survey, there were 175 serious and 205 mild seafood poisonings after consumption of fish, shark, and turtle meals during the period of 1930 to 1996. Sharks were responsible for the most serious poisonings (48%), in addition to other fishes (37%) and marine turtles (11%). Neurological and gastrointestinal features predominated in shark poisonings. Robinson et al. [46] reported on a KAP survey concerning seafood poisonings conducted in Tulear Province with 41 villages spread along 300 km of coast with some 34,000 inhabitants.

Apart from the case reports and outbreaks highlighted above, Ramialiharisoa and colleagues [39] reported on a case series of 10 cases of envenomation by two spiders of the* Latrodectus* genus treated in the Intensive Care Unit of Antananarivo Hospital. In this study which spanned from March 1991 to July 1992, the case fatality rate was 10%.

It is worth noting that there was no literature found on other causes of poisoning apart from those of natural origin.

### 3.8. Malawi

WHO has estimated that there were 0.9 deaths per 100,000 persons due to unintentional poisoning in 2004 [4]. Epidemiological and other data concerning poisoning in Malawi is very limited. Apart from a case report of 12-day-old baby who survived an episode of organophosphate poisoning [48], we managed to identify only three other studies. The first was by Chibwana and colleagues [49] who conducted a one-year prospective study looking at children admitted with poisoning to Queen Elizabeth Central Hospital. They reported a total of 144 admissions of which 118 (82%) were accidental. The case fatality rate in the study was 7.6% with six of the 11 deaths resulting from traditional medicine intoxication. Yu and colleagues [51] reported that poisoning accounted for 15.1% of child injury using medical records at a central referral hospital in Malawi. Dzamalala and colleagues [50] did a retrospective audit of suicides autopsied at Queen Elizabeth Central Hospital and University of Malawi College of Medicine mortuaries between January 2000 and December 2003. Of the 84 suicide cases, the major mode of suicide was chemical poisoning using an agricultural pesticide (*n* = 66; 79%).

### 3.9. Mauritius

WHO has estimated that there were 0.1 deaths per 100,000 persons due to unintentional poisoning in 2004 [4]. Glaizal and colleagues [52] reported on 4 cases of French tourists who suffered ciguatera or ciguatera-like poisoning after visiting Mauritius. Apart from this case report, there was no other data in the medical literature of poisoning in the country.

### 3.10. Mozambique

WHO has estimated that there were 3.4 deaths per 100,000 people due to unintentional poisoning in 2004 [4]. In Mozambique all published literature available to us on poisoning was related to cyanide poisoning resulting from ingestion of cassava, a condition also known as Konzo. Cliff et al. [54] reported an incidence of 34 cases of this poisoning per 1000 in Acordos de Lusaka village, Memba District, in 1981. Casadei et al. [55] reported an incidence of 4 cases per 1000 in Acordos de Lusaka village, Memba District, in 1982. The Mozambique Ministry of Health [53] reported on what was perhaps the first recorded epidemic of Konzo in Mozambique. This work was done in the Nampula province of the country. Briefly, after reports of an epidemic of spastic paraparesis, the Ministry sent out mobile brigades of paramedical workers to search actively for these cases in the province. The brigade members were mostly sanitary agents or nurses and travelled by motorcycle or on foot. They began active detection in August until end of October by which time the epidemic was over and there were few new cases. Active case detection was based on close collaboration with community leaders who were asked to identify patients with difficulty in walking since the last rainy season. The main findings in the study were that there were 1102 patients identified, with the highest incidence rate being 34 per 1000 inhabitants in one village. From the results, 65% of the notified cases were children under 15 years, with males predominating. At over 15 years, females predominated with most cases aged between 20 and 40 years. Although most of the women were lactating, there were no cases of the disease among their babies.

Cliff and Coutinho [56] reported on an epidemic of acute intoxication resulting from ingestion of a newly introduced cassava during drought in Mozambique. This study was done at a Provincial Hospital in Chimoio during the period June 1992–August 1992. The authors reported on a total of 70 cases, of whom 74% were children under 15 years; 17% were women and 9% men. There was one death in a 3-year-old thought to be due to aspiration pneumonia during coma.

Cliff et al. [57] reported on an epidemic of symmetric paraparesis associated with cassava consumption and cyanide exposure (Konzo). This study was conducted in Mujocjo, Nacacana, Moconi, and Terreni A Chieftaincies in Mogincual district, Mozambique. In carrying out the study, community leaders were requested to call patients for examination in all sites except Moconi. Patients were interviewed and given a basic neurological examination. Konzo was diagnosed when patients had a symmetrical spastic abnormality of gait without signs of spinal disease. A priest was also asked to include the Christian community. Urine samples were also collected for thiocyanate, linamarin, and inorganic sulphate measurements from the first 30 children who appeared in each site. The study was conducted in July 1993. The main finding was that 72 patients were diagnosed with konzo. The highest prevalence rate was 30/1000 in Mujocojo Chieftaincy. Of the 72 patients, all had eaten bitter cassava; 89% were between 5 and 44 years. Males predominated in children under 15 years (60%) and women in adults (59%). There were no cases in children below 4 years.

Cliff and colleagues [58] also reported on a project where they examined 397 children for ankle clonus in three districts previously affected by konzo. The study found that the proportion of children with clonus ranged from 4% to 22%. Geometric mean thiocyanate, linamarin, and inorganic sulphate concentrations were 163 and 60 *µ*mol/L and 4.4 mmol/L, respectively.

The last study that was identified in the literature was that of Ernesto and colleagues [59] who examined all schoolchildren in three communities in Memba and Mogincual Districts for ankle clonus. The proportion of schoolchildren with ankle clonus varied from 8 to 17% and 27 new cases of konzo were found. Of these, 17 were children, eight were women, and the remaining two were men. Cassava flour samples were found to contain 26 to 186 ppm of cyanogen. Mean concentrations of urinary thiocyanate in schoolchildren ranged from 225 to 384 *µ*mol/L.

### 3.11. Rwanda

WHO has estimated that there were 1.3 deaths per 100,000 persons due to unintentional poisoning in 2004 [4]. Save for a newspaper article reporting on an outbreak of food poisoning after a Seventh Day Adventist church gathering, there is no literature on the epidemiology of poisoning in Rwanda. In the above case one news agency reported that at least 205 members of the Seventh Day Adventist Church in Gasaka Sector, Nyamagabe District, were receiving treatment at various health centres in Kigeme after consuming contaminated food [117]. Another news agency reporting on the same episode reported 50 admissions [118].

### 3.12. Seychelles

There is no WHO estimate for deaths due to unintentional poisoning [4]. There was only one study for Seychelles by Lagraulet [60] who reported on ichthyotoxism (poisoning by a fish toxin) in the Seychelles Islands. Apart from the aforementioned, the only other publications found in the literature related to pre- and postnatal exposure to methylmercury and the Seychelles Child Development Study, for example, Myers et al., 2009 [61].

### 3.13. Uganda

WHO has estimated that there were 11.4 deaths per 100,000 people due to unintentional poisoning in 2004 [4]. In 1969, Bwibo [62] described cases of accidental poisoning in children based on data from a children's ward of New Mulago Hospital for the period of January 1963 to December 1968, inclusive. From the study, a total of 130 children were admitted with accidental poisoning of which seven died (case fatality rate: 5.4%). The admission rate for the study period was 0.65%. Household chemicals which included kerosene, pesticides, and other poisons used in homes and gardens accounted for the largest proportion of admitted cases (43.1%). For the household chemicals, kerosene was the leading cause of accidental poisoning. With respect to patient demographics, almost all the cases occurred in children under 5 years old (93.1%), with more males (58%) than females. Of the seven deaths, 5 occurred in boys. All the deaths but one were as a result of medicaments.

Cardozo and Mugerwa [63] described the pattern of acute poisoning among Ugandans based on figures obtained from admissions of these cases to Mulago Hospital in Kampala, Uganda. This was a retrospective survey of all cases of acute poisoning admitted during a one-year period, January to December, 1970. The authors looked at all cases admitted to the general wards, but excluded all cases of alcohol poisoning in adults, as well as snakebite and bee stings. There were a total of 70 cases admitted during the study period of which 48 were in children aged 10 years and below. Of these children all the cases were as a result of accidental poisoning with most of the admissions resulting from kerosene ingestion (35.4%) and pesticides accounting for 18.8% of the cases and medicaments only 3 cases. The children represented 0.75% of total paediatric admissions for the period. In adults, almost half of all the cases resulted from pesticide exposure (10 cases). For the adult cases, there was no difference in gender distribution overall. This was 0.35% of the total admissions in adults.

Lubwama [119] reported on five cases of human salmonella food poisoning in Uganda.

Malangu [65] did a retrospective study of acute poisoning cases admitted to two hospitals in Kampala, Uganda, for the period of January 2005 to June 2005. From this study, the author found a total of 276 patients who were admitted to the hospitals. From the cases seen, the mean age was 26.6 years with 71% being males. From the work, agrochemicals (42.4%) were responsible for most of the admitted cases that presented for treatment, followed by household chemicals (22.1%), carbon monoxide (20.0%), snakebites (14.1%), and food poisoning (1.4%). The overall case fatality rate was 1.4% with 75% of those who died being of the male gender. Alcohol accounted for half of all the deaths with carbon monoxide and organophosphate pesticides accounting for 25% each.

Kinyanda and colleagues [64] conducted a case-control study in which 100 cases of deliberate self-harm (DSH) and 300 controls matched on age and sex were recruited from three general hospitals in Kampala. In order to obtain their data, they utilised a structured interview using a modified version of the European Parasuicide Study Interview Schedule 1. From their study they found that poisoning was the most important method used in DSH (65%). Of this category, organophosphate pesticides accounted for the highest proportion (45%) with medications accounting for (35%)—especially diazepam and chloroquine. From their work, they found that pesticides were the preferred method of DSH among males, while medications and other poisons were the preferred methods among females.

Several media reports indicate a high prevalence of methanol poisoning in Uganda. In 2009, the Minister of Health issued a press release on methanol poisoning within the country following a series of deaths that were due to unknown causes in different parts of the country. Following investigations which included interviews with relatives of the deceased and analysis of blood samples of survivors, it was found that a total of 27 people were affected throughout the country and each had ingested alcohol packed in sachets. Nineteen of these individuals died and blood levels of the survivors showed high levels of methanol [66]. Several other deaths have been reported through the media, with 89 people from Kabale and Kamwenge districts in southwest Uganda confirmed dead allegedly due to methanol poisoning in 2010. A further 100 people were hospitalised, including a two-year-old child [67].

### 3.14. United Republic of Tanzania

WHO has estimated that there were 6.6 deaths per 100,000 people due to unintentional poisoning in 2004 [4]. Data relating to poisoning in the published literature is also limited for Tanzania. A study by Rwiza [68] reported on a case series of ten agitated and psychotic patients with other classical signs of atropine poisoning who were admitted to Usangi hospital, Tanzania, after ingesting stiff-porridge made from millet which had been contaminated with seeds of* Datura stramonium* (Jimson weed). All the patients were treated and survived the episode. In this particular series, 7 of the patients were from the same family while the other three lived in separate homes. In the paper, the author alludes to the fact that records of the Chief Government Chemist reported that a similar type of food poisoning had occurred in at least eight other regions over the preceding seven years. The clinical presentation and management of poisoning from* Datura stramonium* are discussed in this paper.

In another study, Yates and colleagues [69] prospectively reviewed patients who, between April 2007 and the end of 2009, received treatment for snakebite envenomation at the Snake Park clinic in Meserani, Tanzania. The authors reported on 85 cases. The mean age was 23 years with a male to female ratio of 1.4. Most of the bites (77%) occurred after dark and during the rainy season (88%). In 32 of the cases, identification of the culprit snake responsible for the bite was not possible. Forty-two cases received antivenom. The case fatality rate was 1% (1 death in a 12-year-old). The authors also reported that 7% of the cases needed a skin graft or amputation of a limb or digit. In cases where the culprit snake was identified, the puff adder was identified in most of the cases.

A hospital based case review by Mbakaya et al. [32] indicated that poisoning due to organochlorine use accounted for 736 cases of poisoning in Tanzania between 1988 and 1990.

A few studies have been published on poisoning related to ingestion of cassava in Tanzania. In the first outbreak, Howlett and colleagues [70, 71] reported 39 cases of Konzo out of the 50 people clinically examined. Thirty of these were male and nine were female aged between 4 and 46 years. Nineteen of the cases were from six families with another 5 cases all from one family. Further investigation of this outbreak revealed 116 cases and 2 deaths due to Konzo. Mlingi and colleagues [72] report on 3 cases of Konzo that occurred in 1988 in Mtanda village, south of Tanzania. Mlingi and colleagues [73] further report on outbreaks of Konzo that occurred in Tanzania in 2001–2003. During this period, twenty-four cases of Konzo occurred in Mbinga District, Ruvuma region (2001-2002), while 214 konzo cases occurred in Mtwara Region in 2002-2003.

A prospective hospital based study of suicides in Dar Es Salaam, Tanzania, indicates that poisoning is a common method of committing suicide with 69% of subjects employing this method [74]. In this study, 28% of subjects used antimalarials (mostly chloroquine) to poison themselves, while 12% of subjects poisoned themselves with pesticides (chlorfenvinphos and diazinon). In the latter, chemicals were identified in 8 out of the 12 cases. The source of poisoning in this study could not be identified for 29 of the subjects.

### 3.15. Zambia

WHO has estimated that there were 4.8 deaths per 100,000 persons due to unintentional poisoning in 2004 [4]. Bhushan and coworkers [76] reported on a retrospective analysis of 378 cases of accidental poisoning by ingestion or inhalation in children admitted to the University Teaching Hospital, Lusaka, Zambia, in 1978. The authors reported a case fatality rate of 0.5%. Paraffin poisoning accounted for the largest proportion of admissions (57.1%), followed by food poisoning (18.3%), household poisons (11%), and medicines (10.8%). Sinclair and colleagues [78] also highlight organophosphorus poisoning as one of the key causes of nontraumatic coma in Zambia.

Gill [75] presented a case series of fourteen cases of mushroom poisoning which presented to the hospitals of Chingola and Chililabombwe on the Zambian copper belt during the period from December 1975 to January 1978. In this case series, 2 patients died, making the case fatality rate 14%. In a combined retrospective and prospective 4-year study of 6412 children consecutively admitted to St. Paul's Hospital, Nchelenge, northeast Zambia, Gernaat and colleagues [77] found that snakebite was a significant cause of injury in 4–14-year-olds.

### 3.16. Zimbabwe

WHO has estimated that there were 8 deaths per 100,000 people due to unintentional poisoning in 2004 [4]. For Zimbabwe a number of papers have been published in the area of poisoning. In order to make for easy reading, the literature concerning poisoning in Zimbabwe is separated into various sections related to the type of group covered by the study under separate headings.

#### 3.16.1. Published Work on Epidemiology of Poisoning in General

By the time of the review, three studies had been conducted in an attempt to describe the overall patterns of poisoning in Zimbabwe regardless of the toxic substances involved and most of what is known today concerning poisoning in Zimbabwe emanates from these two studies. The first was done by Nhachi and Kasilo [79] and this was a retrospective study that looked at all poisoning admissions to six major referral hospitals in the country over a ten-year period (January 1980–December 1989) [79]. The hospitals that were involved included Parirenyatwa, Harare Central, United Bulawayo, Mpilo, Mutare, and Gweru hospitals. Of a total of 6018 cases of poisoning evaluated, the main agents associated with acute poisoning admissions to the hospitals were traditional medicines (22.9%), household chemicals (18.8%), snake and insect envenomation (17.1%), orthodox medicines (16.7%), and insecticides (14.8%). The overall case fatality rate in this study was reported to be 15% with the main agents associated with fatality being pesticides, traditional medicines, and orthodox medicines in descending order.

Tagwireyi and colleagues also did a retrospective case review and looked at all poisoning admissions to eight major referral hospitals in Zimbabwe over a two-year period covering the years 1998-1999 [80]. The study sample for this work included all the hospitals covered by Nhachi and Kasilo, but also added two other referral hospitals, that is, Bindura and Gwanda Hospitals. In this work, the authors reported a total of 2764 admissions resulting from toxic exposure to different toxins and toxicants. They reported a smaller proportion of poisoning cases resulting from accidental exposure (48.9%) than that reported by Nhachi and Kasilo (61.2%; [79]). In addition the spectrum of agents responsible for most admissions had changed with pesticides (32.8%) and pharmaceuticals (20.2%) accounting for most admissions. Household chemicals (15.7%), animal envenomation (11.8%), and natural toxins (6.8%) were also important causes of poisoning. In addition, the case fatality rate from this study which was 4.4 deaths per 100 admissions was much lower than that reported by Nhachi and Kasilo [79]. The work by Tagwireyi and colleagues [80, 89, 91] showed that the patterns of poisoning in Zimbabwe had changed over the last decade or so, with the authors attempting to explain some of these differences.

Tagwireyi and colleagues [81] also reported on the epidemiology of poisoning admissions to six district hospitals in one province of Zimbabwe. The authors compared the epidemiology at these health centres with that of the Provincial hospital serving the districts. They found that the patterns of poisoning were not the same in terms of toxic agents responsible for most cases of poisoning. The case fatality rates were however similar for the district hospitals and the provincial hospital at 4.8% and 4.7%, respectively.

#### 3.16.2. Poisoning in Children

Concerning poisoning in children, Kasilo and Nhachi [86] presented and analysed a total of 2873 cases of children aged between 0 and 15 years. From this report, the authors found that the majority of the cases of childhood poisoning were accidental in nature (93.2%) with most cases occurring in the 0–5-year age group (75.4%). They also reported a case fatality rate of 4.9% with most of the deaths resulting from suicide among the 11–15-year age group and accidental poisonings among the 0–10 years old group. From their study, most of the poisoning patients were male (53.1%). The commonest toxic agents involved included household products (27.2%), traditional medicines (23%), venoms from snakebites and insect stings (16%), and therapeutic agents or pharmaceuticals (12.4%).

Chitsike [92] reported on a retrospective study of forty-two cases of acute poisoning admitted to a paediatric Intensive Care Unit at Parirenyatwa over a two-year period (1990-1991, inclusive). This study was different to that conducted by Kasilo and Nhachi in that it looked only at the severe cases of poisoning in children who required intensive care. However, as was the case with the former study, Chitsike also found that household products as exemplified by paraffin (26.2% of cases), traditional medicines (14.3%), and pharmaceuticals were the commonest causes of poisoning in children. As expected from the intensive care unit, the case fatality rate in the series by Chitsike was much higher than that reported by Kasilo and Nhachi [86], being 21%.

Tagwireyi and colleagues [91] reported on a total of 761 cases (aged 0–12 years) of childhood poisoning admitted to eight study hospitals of which 97.5% (742) were accidental with the majority of the cases (>80% of all childhood poisoning admissions) occurring before the age of 5 years. In addition, over 56.0% of the cases occurred in males. Cases of deliberate self-poisoning occurred in children aged 10 years and above, similar to the study by Kasilo and Nhachi [86]. The main agents responsible for poisoning admissions were household chemicals, especially paraffin (43.2%), pesticides (23.1), natural toxins which included traditional medicines (13.9%), and animal envenomation which included snakebite and scorpion sting (11.6%). The case fatality rate in this work was 3.1%.

#### 3.16.3. Pesticide Poisoning

Zimbabwe has a largely agrarian based economy and consequently depends heavily on the use of pesticides. However, despite the importance of pesticide poisoning in the country, there is sparse data relating to important epidemiological characteristics of pesticide poisoning with only a handful of studies having been published. The first report pertaining specifically to pesticide poisoning after independence in 1980 was done by Nhachi [84]. In this work, Nhachi compared a total of 161 cases of organophosphate poisoning cases admitted to Parirenyatwa hospital (for the period from January 1981 to June 1986) with 30 cases recorded at Shamva hospital (over 11 months). The study revealed that while the bulk of cases admitted to Parirenyatwa hospital were intentional poisonings (83% of cases), those admitted to the rural hospital were mainly accidental (70%). The author attributed this to “social factors” as exemplified by greater social pressures resulting from urbanisation. The case fatality rate for cases admitted to the urban hospital was 14% whereas the rural hospital did not record any fatalities during the 11-month study period.

Later, Kasilo and coworkers [83] published a subanalysis of a larger study on the pattern of poisoning in urban Zimbabwe [79]. This work described the pattern of organophosphate poisoning in Zimbabwe and found that these pesticides accounted for 10.1% (606 cases) of all the admissions to their study hospitals with most of these cases resulting from deliberate self-poisoning (75%), and 21% being accidental. The case fatality rate was 8%. The authors also reported that most admissions occurred in the 21–30-year age group (42% of all cases).

Dong and Simon [82] carried out a study to examine organophosphate poisoning in Zimbabwe by determining the trends of organophosphate admissions in an urban Zimbabwean hospital. Over a period of six years (1995 to 2000), they found 599 cases of organophosphate exposure, of which the male and female admission rates were similar (48% were male). The authors reported that suicide was the predominant cause of organophosphate poisoning accounting for 74% of the admissions. The case fatality rate in the series was reported as 8.3%. Dong and Simon [82] concluded that organophosphate poisoning is increasing rapidly with an increase in admissions of 320% over the six years of the study.

More recently, Tagwireyi and colleagues [115] arguing in the introduction to their paper that too much emphasis had been placed on individual pesticides, especially organophosphates in all past publications, reported on the epidemiology of pesticide poisoning in general, regardless of pesticide type. The authors found that of the 914 single pesticide exposure in their study, almost half (49.1%), resulted from oral exposure to rodenticides and 42.2% from anticholinesterase-type pesticides (AChTP), mostly organophosphates which were responsible for over 90% of admissions from AChTP. Accidental and deliberate self-poisoning (27.1% and 58.6%, resp.) accounted for most cases with only 8 homicides. The case fatality rate (CFR) in deaths/100 admissions was 6.8%. They revealed an important aspect pertaining to pesticide poisoning, which had not been highlighted before, that most of the cases of pesticide poisoning now resulted from exposure to the illegal rat poisons, popularly known as “mushonga yemakonzo,” which were being sold in street corners in Zimbabwe. The authors from their results recommended that stricter control should be done of these substances.

#### 3.16.4. Pharmaceutical Poisoning

Concerning poisoning from orthodox medicines, Nhachi and colleagues [111] carried out a subanalysis of their data on the patterns of poisoning admissions to major referral hospitals in Zimbabwe. The authors reported that pharmaceutical poisoning admissions to the study hospitals resulted from mainly accidental exposure (63.5%). In addition, analgesics (22% of all pharmaceutical admissions), sedatives and hypnotics (13.2%), antipsychotics (11.6%), and antimalarials (9.3%) were the major groups of drugs implicated, with the case fatality rate in the study being 3.9 deaths per 100 admissions (3.9%).

Queen and colleagues [112] reported on an increasing incidence of poisoning admissions involving chloroquine, suggesting that the pattern reported by Nhachi and coworkers may have changed. The authors looked at the pattern of serious chloroquine overdose based on a retrospective examination of all toxicology cases recorded at the Parirenyatwa Hospital Casualty Department resuscitation room for the period from May 1, 1987, to April 30, 1995. They found a statistically significant rise in the number of chloroquine overdose cases presenting to the resuscitation room during the study period from nil cases in 1987/1988 to 33 in 1994/1995 (*p* = 0.001), with a case fatality rate of 40%. In addition, a preponderance of females taking chloroquine in overdose, compared to other overdoses and toxic exposure, was reported (OR 1.99; 95% CI 1.31–3.04; *p* < 0.001). Queen and colleagues [112] postulated that this gender distribution could have been related to the use of high doses of chloroquine to induce abortion and recommended further investigation into this issue.

Another study looking specifically at aspects of poisoning from pharmaceuticals was done by McKenzie [113]. The author described the features of chloroquine poisoning by carrying out a retrospective review of all cases of confirmed acute chloroquine poisoning admitted to intensive care units at Harare and Parirenyatwa central hospitals for the period from November 1990 to October 1994. Of the 29 cases identified, 69% (20) were female and 31% (9) were male with a case fatality rate of 20.7%. The common clinical features in the patients were similar to those documented in the literature and included respiratory failure, depressed level of consciousness, hypothermia, hypotension, cardiac arrest, and hypokalaemia.

Ball and colleagues [114] reported on a retrospective hospital record review to describe the epidemiology of chloroquine poisoning compared with that of other medicines. They selected all records of admissions to the eight referral hospitals due to poisoning with single pharmaceutical agents and separated these into those involving either chloroquine or other medicines. Case characteristics were compared and a retrospective cohort study was performed to investigate the association of pregnancy with chloroquine overdose. From their analysis, of 544 cases, antimalarials accounted for the largest proportion of admissions (53.1%), with chloroquine accounting for 96.2% (279 cases) of these. The cases of chloroquine poisoning were then compared with the remaining 265 cases. The authors found that a greater proportion of patients took chloroquine deliberately (80.3% versus 68.7%; *p* < 0.05) and that case fatality rate due to chloroquine poisoning was significantly higher than that of poisoning due to other drugs (5.7% versus 0.7%; *p* < 0.0001). They also found that patients admitted with chloroquine poisoning (188 cases) were twice as likely to be found pregnant (relative risk = 2.3, 95% CI = 1.2–4.5) than similar women admitted due to other medicines (157 cases). The authors concluded that chloroquine is the most common cause of pharmaceutical poisoning admission at referral hospitals in Zimbabwe.

#### 3.16.5. Household Chemical Poisoning

Concerning household chemical poisoning, Nhachi and Kasilo [98] carried out a retrospective analysis to evaluate the epidemiology of poisoning by household chemicals based on their data on admissions at Zimbabwe's six main urban hospitals over a 10-year period (1980–1989). They reported that a total of 1192 household chemicals poisoning cases were recorded, and this constituted a fifth of all poisoning cases recorded during the study period. In line with international literature on the subject, the researchers found that the majority of the cases (61.3%) occurred in the 0–5-year age group, with the 16–25- and 26–30-year age groups accounting for 11% and 5.2%, respectively. The authors also reported as expected that the bulk of the cases (66.8%) resulted from accidental exposure to the chemicals. They reported a case fatality rate resulting from household chemical exposure of 13% with most of the deaths being suicides. Paraffin (kerosene) was the most common poisoning agent accounting for 68% of the cases. This was followed by rat poisons (5.8%), bleaches (5.1%), and caustic soda (3.3%). The authors concluded that incidence of poisoning with household chemicals could be reduced by health education directed to parents emphasising the importance of safe storage of paraffin and other household chemicals and by legislation to stop retailers from selling paraffin for domestic use in second-hand containers.

More recently, Tagwireyi and coworkers [103] reported on a total of 327 admissions due to oral exposure to paraffin which represented 11.8% of all the poisoning admissions to the eight study hospitals. In accordance to literature from other countries, most exposure instances (91.7%) occurred accidentally, with only 6.7% resulting from deliberate ingestion of the chemical. Over 85% of cases were in the 0–5-year age range and less than 10% were above the age of 12 years. The median age on admission was found to be much higher for deliberate self-poisoning (23 yrs; IQR 19–26 yrs) compared to that for accidental poisoning (1.5 yrs; IQR 1-2 yrs). The authors also examined the drug management of paraffin poisoning and found that over three-quarters of patients received an antibiotic either alone or in combination with another antibiotic or drug. Paracetamol (24.3%) was the next most commonly encountered treatment. The case fatality rate was 0.3 deaths per 100 admissions (95% Confidence Interval 0.0–1.7).

#### 3.16.6. Animal Envenomation—Snakebite

Concerning snakebite, there have only been a handful of studies on the epidemiology and other related factors of snakebite, most of which have been based on data from single hospitals or case reports. Blaylock [93] described various clinicoepidemiological aspects of this type of envenomation based on clinical observations from retrospective data of 250 snakebite cases admitted to a hospital in the south-eastern lowveld of Zimbabwe (Triangle hospital) over six and a half years (January 1975 to June 1981). He presented data on the clinical presentations, types of snakes, and treatment given to snakebitten patients attending the hospital with a case fatality rate of 0.4%.

Geddes and Thomas [94] reported on a case report of a boomslang bite in a 30-year-old snake-handler who was bitten around the shoulder blade area. This patient was successfully treated with the South African Institute of Medical Research (SAIMR) monovalent boomslang antivenin.

Kasilo and Nhachi [95] reported on the pattern of snakebite in Zimbabwe from 995 cases of snakebite. The authors noted a mortality rate of 1.8% in the series as well as the fact that antibiotics were the most commonly used treatment for snakebite and were often used irrationally. In addition, they reported that most snakebites occurred in patients aged between 16 and 20 years. The authors reported that the few records of the types of snakes associated with envenomation were, in order of frequency, cobra, adders (puff and night), mamba, and boomslang. They concluded from their results that prevention and prompt treatment of snake envenomation were a priority for the reduction of incidence of poisoning.

As no studies had been done in the past reporting on snakebite admissions to rural/district hospitals where one would expect it to be rife, Nhachi and Kasilo [99] reported a series of 274 cases of snakebite admitted to hospitals in the eight provinces of Zimbabwe. This was and is the only prospective study on snakebite conducted in Zimbabwe in the area of clinical toxicology. This study was done over a period of 2 years (January 1991 to December 1992). From this work, only five deaths (1.8% of the total cases) were reported. The majority of snakebites (63%) occurred at night (between 6.30 p.m. and midnight) and over 74% took place during the hot rainy season. Of the snakes identified in the bites, the cobra was identified in 37% of cases, the puff adder in 20% of cases, and the black and green mamba in 18% of the cases. In line with their earlier study, Nhachi and Kasilo noted that treatment of snake envenomation consisted mainly of the administration of antibiotics (151 cases). Analgesics (144 cases), antivenom tropical snake polyvalent (ATT) (89 cases), antitoxoid tetanus (TT) (61 cases), antihistamines (47 cases), and traditional medicines (43 cases) were also used.

Muguti and colleagues [97] reported a retrospective analysis of 83 consecutive patients treated for snakebite at one of the central hospitals in Zimbabwe (Mpilo) for the period from January 1990 to June 1992. In this study, the authors also reported that antibiotics were the most commonly used medication for snakebite, in addition to presenting data on the most frequently bitten areas and clinical features. The mortality rate was 5%, which was attributed to the lack of antivenom at the hospital during the study period [97]. Muguti and Dube [100] published what is perhaps the only published clinical case report of a bite from the vine snake* (Thelotornis capensis oatessi)* in Zimbabwe for which antivenom was not available [100]. In this case report, the patient survived the bite after vigorous supportive therapy.

Tagwireyi and colleagues also recently reported on the patterns and epidemiology of snakebite admissions to the eight major referral hospitals included in their main study [101]. In their work, these researchers reported on a total of 273 snakebite admissions. Of these the type of snake involved in the bites was recorded in 14.6% of the cases with 62.5% involving puff adders and 22.9% involving cobras and mambas. As with all other studies on snakebite in Zimbabwe, the gender distribution was similar. Again, in line with reports from other studies in Zimbabwe, most of the bites (>80%) occurred during the summer months of November to April. The case fatality rate from snakebite was also comparable to earlier studies being 2.9%. These results were recently later published in a full paper by the same authors [102].

#### 3.16.7. Animal Envenomation—Scorpion Sting

Saunders and Morar [106] reported on a case of scorpion envenomation, thought to have resulted from a scorpion of the genus* Parabuthus* judging from the clinical picture of the patient. In their case report the patient survived without any specific scorpion antivenin administration. In a ten-year retrospective study of all cases of poisoning to six major referral hospitals in Zimbabwe, Nhachi and Kasilo [107] reported that only five cases of scorpion sting were admitted to the study hospitals, with no further details on the cases. They also reported that, of the 92 cases of insect and scorpion sting/bite admissions, bees (44.6%), wasps (8.7%), and spiders (8.7%) accounted for most of the exposure instances. No fatalities were recorded in this series indicating that scorpion sting and insect bites were rarely fatal in Zimbabwe.

Bergman [104] described the epidemiological and clinical features of scorpion stings in Gwanda South District. He collected a case series of consecutive scorpion sting victims presenting to Manama Hospital and all seven rural health centres in Gwanda South District, Zimbabwe, between September 1991 and September 1993. Bergman reported 244 cases, of which 184 were* Parabuthus transvaalicus*, Purcell, 1899. From this work, the author identified seventeen patients with severe* P. transvaalicus* scorpionism and published a case series of these patients and their clinical picture [105]. From this study, Bergman described the clinical features of* P. transvaalicus* scorpionism for the first time. He noted that they resembled those of* P. granulatus* scorpionism which, however, has significant sympathetic nervous system stimulation, the distinguishing features being visual disturbances, anxiety, restlessness, and raised blood pressure. The case fatality rate was 0.3% in the study by Bergman and mortality rate in the district was 2.8 per 100,000 per year. Tagwireyi and Ball [108] also reported on a case series of scorpion sting admissions to major referral hospitals in Zimbabwe. In this paper, there were no fatalities reported.

#### 3.16.8. Traditional Medicine Poisoning

Nyazema [85] carried out a study to determine the number of cases of poisoning due to traditional medicines as recorded at Parirenyatwa, Harare Central hospitals, and the Government Analyst Laboratory. The author reported a total of 297 cases admitted to Harare hospital for the period from 1971 to 1982. Specific numbers were not given in the text for the number of cases recorded at the other two institutions. Moreover, although the author stated in his paper that the mortality at Harare hospital increased twofold, no specific figures were given. Nyazema [120] also reported on results of a study of the records of four hospitals from 1971 to 1982, carried out to see how many people had been poisoned with herbal remedies. As one of the main findings, the author noted that the number had increased since 1971. In addition, the author interviewed a total of 50 traditional healers concerning record-keeping and knowledge of toxicity. Kasilo and Nhachi [87] then reported on a subanalysis of a total of 1456 cases of traditional medicine poisoning from their 10-year retrospective study of all poisonings. They reported that poisoning from traditional medicines represented 23% (the biggest single group) of all poisoning cases. Almost two-thirds (67%) of the patients were male and most of the admitted patients were under 5 years of age (53%). Of the traditional medicine poisoning cases, 61% of poisonings were associated with treatment of an ailment and 8% were accidental poisonings, while 2.2% resulted from deliberate self-poisoning. The authors reported a case fatality rate of 6% from their study. They found that the main reasons for seeking treatment with traditional medicines were for depressed fontanelle and fever in children and diarrhoea and abdominal pain in adults. Treatment in all poisoned patients consisted mainly of supportive therapy and included the induction of vomiting with ipecacuanha in children.

Tagwireyi and Ball [88] after noting a paucity of information regarding the clinical presentation of poisoning associated with use of traditional medicines (“muti”) in Zimbabwe, conducted a small case series describing the common adverse effects of poisoning and relating these with the reasons for taking the traditional medicines. They looked at all records of admissions for traditional medicine poisoning to Parirenyatwa hospital over the period 1995 to 1999, inclusive. From a total of 16 cases, Tagwireyi and Ball reported that the main reason for taking traditional medicines was to treat abdominal pain (five cases). They also found that, of these five cases, most suffered from toxic effects related to the genitourinary system, mainly dysuria (4 of 5 cases) and haematuria (5 of 5 cases). Due to the small numbers in their series, the researchers suggested that further studies were needed to ascertain if “muti” taken for abdominal pains contains a nephrotoxic agent.

Tagwireyi and colleagues [89] then followed up on their earlier study by carrying out a retrospective review of all cases of poisoning with traditional medicines at eight main referral hospitals in Zimbabwe (January 1998–December 1999, inclusive) to describe the most common signs and symptoms, reasons for taking the medicine, and management of poisoning in adults. From a series of 63 cases, they found that in line with their earlier work, where the reasons for taking the traditional medicine were known, most cases had taken it for either abdominal pains or aphrodisiac purposes. Nonspecific adverse effects including vomiting, abdominal pains, and diarrhoea were the most commonly encountered. Again in line with their earlier work, Tagwireyi et al. showed that a large proportion of patients with traditional medicine poisoning also suffered from genitourinary tract adverse outcomes especially haematuria and dysuria. In their work the authors speculated that the nephrotoxic effects seen could be due to a number of things including heavy metal contamination of traditional medicines, cantharides, and inherent nephrotoxic effects of the phytomedicines themselves. The authors suggested that further research was required to elucidate the toxic components responsible for the observed ill effects and whether these effects are due to the medicines themselves or to coexisting illnesses. The case fatality rate in this series was 9.5%. The exact culprit agents associated with deaths were not identified.

#### 3.16.9. Plant and Mushroom Poisoning

In view of “a large proportion” of calls to the national Drug and Toxicology Information Service as well as a lack of published data on the toxicoepidemiology and management of Elephant's Ear plant (which is in the Araceae family of plants), Tagwireyi and Ball [121] did a small retrospective series of all cases of Elephant's Ear ingestion. Their study looked at hospital medical records from Parirenyatwa hospital for the period of January 1995–December 1999, inclusive. In their work, the authors reviewed 15 cases of Elephant's Ear plant ingestion and described the clinical signs and symptoms associated with the exposure. In line with international literature, they also noted that almost all cases occurred in children aged 4 years and below (only one case was in an adult aged 17 years). There were no fatalities in this study.

Mushroom poisoning occurs mainly during the rainy season when these fungi grow. There are a large number of fungi in the world but perhaps the most toxic of these belong to the* Amanita* family of fungi. Flegg [90] reported on 50 cases of mushroom poisoning admissions to Harare hospital for the period from March 1980 to March 1981. As expected, the author found that the bulk of the admissions occurred during the rainy season with 80% of the cases occurring between December 1980 and January 1981. In the paper, the author described the clinical course of all the cases noting that most (80%) of the cases presented with either gastrointestinal symptoms, neurologic symptoms, or both. The case fatality rate in this study was 12% with six deaths. Of these, the postmortem results were available in four and showed evidence of haemorrhage in many organs including the lungs, pericardium, gastrointestinal tract, and urinary tract. In two cases, centrilobular liver necrosis and acute tubular necrosis of the kidneys were present. The author accounted that* Amanita phalloides* had been responsible for all the deaths. The author then went on to describe the identification, toxic chemistry, pharmacokinetics, clinical features, diagnosis,and treatment of poisoning from* Amanita phalloides* mushrooms.

#### 3.16.10. Food Poisoning

Concerning food poisoning, Kasilo and Nhachi [109] reported on 487 cases of food poisoning. In their study, the authors identified mushroom (47%), food-borne, and other related toxins (37%) as the major cases of food poisoning. The case fatality rate in this study was reported as 2.5%. Tagwireyi and colleagues [110] reported a unique case of food poisoning after the ingestion of a poisonous insect* (Mylabris dicincta)*, commonly referred to as the “blister beetle.” They gave a detailed case report of this toxic ingestion in a four-year-old girl whom the authors speculated could have mistaken the beetles for the edible* Eulopidae mashona*. The patient presented with classical signs and symptoms of cantharidin poisoning with blistering in the mouth, hypersalivation, haematuria, and abdominal pains. The authors described the management administered to this patient with the consultation of the national poisons information centre. The patient survived the episode and was discharged after nine days of admission. In their paper, the authors presented an overview of the clinical effects of cantharidin toxicity as well as its treatment.

#### 3.16.11. Poisoning from Metals

Kasilo and Nhachi [96] reported on 40 cases of heavy metal poisoning from their retrospective study of six referral hospitals in Zimbabwe (although it is interesting to note that 5 of these were cases of cyanide poisoning). The authors identified eight different metals as culprit agents in the poisoning cases of which copper accounted for the largest proportion (27.5%). The majority of the cases (70%) were in the 18–60-year age range. From the study it is not clear what the case fatality rate was although the authors stated that 5 cases (12.5%) were suicides (they did not stipulate whether these were attempted/failed suicides or completed/successful suicides).

## 4. Conclusions

The present paper sought to highlight important aspects of the patterns and epidemiology of poisoning in the Eastern African region. From the results presented above, it is evident that pesticides are an important cause of poisoning in the region. In addition, snakebite is also a cause for concern. Also of note was the fact that island countries had patterns of poisoning very different to landlocked countries.

## Figures and Tables

**Table 1:** Literature review of poisoning in the Eastern Africa subregion.

Country	Authors (year)	Study setting	Study period	Type of study	Outcome of interest	Number of cases reviewed	Main results
Burundi	WHO (2009) [4]	National statistics	2004	Burden of disease estimation	Mortality from unintentional poisoning	—	7.8 deaths per 100,000 persons
Comoros	WHO (2009) [4]	National statistics	2004	Burden of disease estimation	Mortality from unintentional poisoning		1.7 deaths per 100,000 persons
Djibouti	WHO (2009) [4]	National statistics	2004	Burden of disease estimation	Mortality from unintentional poisoning		3.9 deaths per 100,000 persons
Djibouti	Benois et al. (2009) [5]	French Military Hospital, Djibouti	18 mths, 2006-7	Prospective descriptive study	Childhood kerosene poisoning	17	11 (64.7%) with pulmonary signs, 7 (41%) with pneumonia, and 6 (35%) asymptomatic
Djibouti	Seignot et al. (1992) [6]	Hopital d'Instruction des Armees		Case report	Snakebite *(Echis carinatus)*	1	Fatal outcome
Djibouti	Larréché et al. (2011) [7]	Intensive care unit of French Military Hospital, in Djibouti	Oct 1994–May 2006	Retrospective case review	Effectiveness of delayed antivenom administration in Snakebite with African viperidae	73	64 (76%) given antivenom; 68 (93%) had coagulopathy; administration of antivenom effective in correcting coagulopathy even if given >24 hours after bite
Djibouti	Aigle et al. (2010) [8]		July 2008–July 2009	Prospective case series	Stingray stings		12 stings treated during study period
Eritrea	WHO (2009) [4]	National statistics	2004	Burden of disease estimation	Mortality from unintentional poisoning		3.7 deaths per 100,000 persons
Ethiopia	WHO (2009) [4]	National statistics	2004	Burden of disease estimation	Mortality from unintentional poisoning		3.5 deaths per 100,000 persons
Ethiopia	Abebe (1991) [9]			Retrospective case review	Organophosphate poisoning	50	Case fatality rate was 20% 94% of cases were attempted suicide
Ethiopia	Aseffa et al. (1994) [10]	Gondar College of Medical Sciences Students Clinic	31 Dec–4 Jan 1992	Prospective case series	Food poisoning *(Salmonella Newport)*	344	79 (23%) of students had clinical symptoms of food poisoning
Ethiopia	Alem et al. (1999) [11]	Butajira rural district	Nov 1994–Jan 1995	Community based cross-sectional survey	Suicide attempts among adults	332	Poisoning (42.4%) was second most common method of attempting suicide; strong detergents and rodenticides most commonly used by women
Ethiopia	Abula and Wondmikun (2006) [12]	Gondar University Teaching Hospital	Jul 2001–Jun 2004	Retrospective case review	Acute poisoning	102	Poisoning accounted for 0.45% of emergency room admissions; organophosphates accounted for 41.5% of poisoning cases; case fatality rate 2.4%
Ethiopia	Melaku et al. (2006) [13]	Tikur Anbessa Specialised Teaching Hospital	1985–2000	Retrospective review of admissions to ICU	Acute poisoning	3548	168 (4.7%) admissions and 44 (3.9%) deaths due to organophosphate poisoning
Ethiopia	Desalew et al. (2011) [14]	Tikur Anbessa Specialised Teaching Hospital	Jan 2007–Dec 2008	Retrospective study	Acute adult poisoning	116	Most (96.5%) of the cases were intentional self-harm cases with household cleaning agents being the leading toxicants used (43.1%) followed by organophosphates (21.6%); the case fatality rate from this study was reported to be 8.6%
Ethiopia	Azazh (2011) [15]	Tikur Anbessa Specialised Teaching Hospital	Jan 2007	Case report	Organophosphate poisoning	1	N/A
Ethiopia	Selassie (1998) [16]	Jimma Hospital	Jan 1996–Jan 1997	Prospective study of admissions	Organophosphate poisoning	23	Male : female ratio was 1 : 2.83; most common clinical findings were vomiting and abdominal pain; no deaths; average time to reach hospital was 18 hours
Ethiopia	Makita et al. (2012) [17]	Debre Zeyit, Ethiopia	N/A	Mathematical modelling	Staphylococcal poisoning	N/A	Authors estimated that the annual incidence rate of staphylococcal poisoning in the area was 20 per 1000 people (90% CI: 13.9–26.9)
Ethiopia	Aga and Geyid (1992) [18]	Clinics	July-August 1984		*Datura stramonium*	688	Case fatality rate was 1.31%
Kenya	WHO (2009) [4]	National statistics	2004	Burden of disease estimation	Mortality from unintentional poisoning		3.4 deaths per 100,000 persons
Kenya	Charters (1957) [19]	Hospital	1949–52	Case reports	Mushroom poisoning	3	Clinical signs and symptoms described
Kenya	Davidson (1970) [20]	Hospital		Case report	Snakebite *(Naja mossambica pallid)*	1	Patient survived
Kenya	Mwangemi (1976) [21]	Wajir District Hospital	Dec 1973–Dec 1975	Retrospective case review	Snakebite	38	Case fatality rate was 2.6%
Kenya	Greenham (1978) [22]	Garissa Provincial Hospital	Nov 1976	Case report	Snakebite	1	Clinical picture of snakebite resulting from the spitting cobra *(Naja mossambica pallida)* in a 5-year-old child
Kenya	Smith et al. (1979) [23]	Gabra nomads	?	Case series	Botulism	300	Attack rate for the entire community of 300 was 3% and 62% for the funeral attenders Case fatality rate among nomads was 50%
Kenya	Kahuho (1980) [24]	Intensive care unit in Kenyatta National Hospital	Aug 1972–Apr 1978	Retrospective case review	Drug and other chemicals poisoning	72	Incidence of 33.7 cases per 1000 admissions Case fatality rate was 36.1% (52% for children under 5 years)
Kenya	Ngindu et al. (1982) [25]	Three hospitals in Machakos district	?	Case series	Aflatoxicosis	20	60% case fatality rate
Kenya	Snow et al. (1994) [26]	Kilifi District, Mombasa	1994	Community based retrospective survey	Snakebite	4712 households	Annual rate of snakebite estimated to be 150 per 100,000 people; only 19% of the victims were bitten by potentially venomous snakes; no deaths
Kenya	Coombs et al. (1997) [27]	(i) Kakamega and western Kenya, (ii) Lake Baringo and Laikipia, (iii) Kilifi and Malindi, and (iv) northern Kenya		Community based cross-sectional survey	Snakebite		The overall average frequency of snakebite was 13.8 per 100,000 people per year and the minimum rate of snakebite mortality was 0.45/100,000/year
Kenya	CDC (2004) [28]	Eastern and central provinces	Apr 2004–Jul 2004	Case series	Aflatoxicosis	317	39.4% case fatality rate
Kenya	Guantai et al. (1993) [29]	19 Kenyan District and provincial hospitals and Kenyatta National Hospital	3 years	Retrospective case review of paediatric poisonings	Poisoning	1904 in total, of which 40% were children <15 years	In the under five years group paraffin, drugs, and organophosphates accounted for 41.09, 23.81, and 15.17% of poisoning cases, respectively
Kenya	Lang et al. (2008) [30]	Kilifi District Hospital	Jan 2005–Dec 2006	Retrospective case review of paediatric poisonings	Accidental paraffin poisoning	48	Incidence of children hospitalised with paraffin poisoning was 17 in 100,000 Case fatality rate was 2%
Kenya	Musumba et al. (2004) [31]	Hospital		Case report	Salicylate poisoning	3	
Kenya	Mbakaya et al. (1994) [32]	Hospital	1998/1990	Retrospective case review	Pesticide poisoning	455	455 cases of organochlorine poisoning
Kenya	BBC news 20 Nov (2000) [33]	Media report	2000		Methanol poisoning	>640 cases	512 poisonings, plus 130 deaths from drinking chang'aa; it is also noted that more than 80 people died in 1998
Kenya	Ministry Environment (2011) [34]	National chemicals profile	2005		Methanol poisoning		50 deaths
Kenya	Nyamu et al. (2012) [35]	Kenyatta National Hospital	Jan 2002 to June 2003	Study of admissions	Poisoning	458 cases	Most common poisoning was due to pesticides, accounting for 43% of admissions, followed by household products at 24%
Madagascar	WHO (2009) [4]	National statistics	2004	Burden of disease estimation	Mortality from unintentional poisoning		2.9 deaths per 100,000 people
Madagascar	Vicens et al. (1986) [36]	Various hospitals	1982	Laboratory investigation of cases of botulism and analysis of suspected food	Food-induced botulism	20	Botulinum toxin Type E identified on bioassay
Madagascar	Domergue (1989) [37]	Hospital		Case reports	Snakebite *(Colubrida opisthoglypha)*	2	
Madagascar	Habermehl et al. (1994) [38]			Outbreak report	Severe ciguatera/ciguatera-like poisoning	>500	Case fatality rate was 20%
Madagascar	Ramialiharisoa et al. (1994) [39]	Hospital	Mar 1991–Jul 1992	Observational	Spider bite (latrodectism)	10	Case fatality rate was 10%
Madagascar	Ramialiharisoa et al. (1996) [40]; Ramialiharisoa et al. (1997) [41]	Vohipeno		Outbreak report	Ciguatera/ciguatera-like poisoning	600	310 patients admitted, 4 deaths
Madagascar	Ranaivoson et al. (1994) [42]		Dec 1994	Outbreak report	Seafood (sea turtle) poisoning	60	The poisoning attack rate was 48% The case fatality rate was 7.7%
Madagascar	Boisier et al. (1994) [43]	Hospital		Prospective observational	Seafood *(Carcharhinus leucas)* poisoning	200	The poisoning attack rate was 100% The case fatality rate was 30%
Madagascar	Champetier De Ribes et al. (1998) [44]	National surveillance	Jan 1993–Jan 1998	Prospective epidemiological study	Seafood poisoning	19 episodes	1789 people poisoned; 70% of episodes were due to consumption of sea turtle or shark; there were 102 deaths (case fatality rate of 6%)
Madagascar	Ribes et al. (1999) [45]	560 villages with 585,000 people along the Madagascar coast	1996-1997	Community based knowledge, attitude, and practice (KAP) survey	Seafood poisoning	380 cases of poisoning recalled over 1930–1996	Sharks were responsible for most serious poisoning (48%), in addition to other fishes (37%) and marine turtles (11%); neurological and gastrointestinal features predominated in shark poisonings
Madagascar	Robinson et al. (1999) [46]	Tulear Province with 41 villages spread along 300 km of coast with about 34,000 inhabitants	Jun-July 1996	Community based KAP survey	Seafood poisoning	84	Cases reported over period 1931–1995, involving fish, sharks, and turtles; case fatality rate of 16.7%
Madagascar	Ravaonindrina et al. (2001) [47]		July 1998	Case series	Puffer fish poisoning	4	One death; tetrodotoxin identified.
Malawi	WHO (2009) [4]	National statistics	2004	Burden of disease estimation	Mortality from unintentional poisoning		0.9 deaths per 100,000 persons
Malawi	O'Reilly and Heikens (2011) [48]	Hospital		Case report	Organophosphate poisoning	1	Survived
Malawi	Chibwana et al. (2001) [49]	Queen Elizabeth Central Hospital	1 year	Prospective observational	Childhood poisoning	144	Most (82%) of admissions were due to accidental poisoning Case fatality rate was 7.6%
Malawi	Dzamalala et al. (2006) [50]	Queen Elizabeth Central Hospital and University of Malawi College of Medicine Mortuaries	Jan 2000–Dec 2003	Retrospective audit of suicides autopsied	Deliberate self-harm leading to death (suicides)	84	Pesticide poisoning accounted for 66 cases (79%) of suicide
Malawi	Yu et al. (2009) [51]	Central referral hospital	?	Retrospective case review	Childhood injury		Poisoning accounted for 15.1% of child injuries in the study
Mauritius	WHO (2009) [4]	National statistics	2004	Burden of disease estimation	Mortality from unintentional poisoning		0.1 deaths per 100,000 persons
Mauritius	Glaizal et al. (2011) [52]	?	March 2010	Case reports	Ciguatera/ciguatera-like poisoning	4	Clinical poisoning, with recurrence 1 year later
Mozambique	WHO (2009) [4]	National statistics	2004	Burden of disease estimation	Mortality from unintentional poisoning		3.4 deaths per 100,000 persons
Mozambique	Ministry of Health (1984) [53]	Nampula province	Aug–Oct	Community based cross-sectional survey	Spastic paraparesis (mantakassa/konzo) caused by cassava consumption	1102	Highest incidence rate was 34 per 1000 inhabitants in one village
Mozambique	Cliff et al. (1986) [54]	Acordos de Lusaka village, Memba District	1981	?	Konzo caused by cassava consumption	?	Incidence rate was 34 cases per 1000 people
Mozambique	Casadei et al. (1990) [55]	Acordos de Lusaka village, Memba District	1982	?	Spastic paraparesis caused by cassava consumption	?	Incidence rate was 4 cases per 1000 persons
Mozambique	Cliff and Coutinho (1995) [56]	Provincial Hospital in Chimoio	Jun–Aug 1992	Case series	Acute cassava intoxication	70	0.14% case fatality rate 74% children under 15 years; 17% women and 9% men
Mozambique	Cliff et al. (1997) [57]	In Mujocjo, Nacacana, Moconi, and Terreni A Chieftaincies in Mogincual district, Mozambique	July 1993	Community based cross-sectional survey	Spastic paraparesis caused by cassava consumption	72	The highest prevalence rate was 30/1000 in Mujocojo Chieftaincy
Mozambique	Cliff et al. (1999) [58]	Mogincual district	July 1993		Ankle clonus, thiocyanate, linamarin and sulphate excretion	397	Proportion of children with clonus ranged from 4% to 22%; geometric mean thiocyanate, linamarin, and inorganic sulphate concentrations were 163 and 60 *µ*mol/L and 4.4 mmol/L, respectively
Mozambique	Ernesto et al. (2002) [59]	Memba and Mogincual districts: 3 communities	Oct 1999	Community based survey	Konzo and cyanogen in flour	27	Proportion of schoolchildren with ankle clonus was 8% to 17%; 27 new cases of konzo were found; cassava flour samples were found to contain 26 to 186 ppm of cyanogen.
Rwanda	WHO (2009) [4]	National statistics	2004	Burden of disease estimation	Mortality from unintentional poisoning		1.3 deaths per 100,000 persons
Seychelles	WHO (2009) [4]	National statistics	2004	Burden of disease estimation	Mortality from unintentional poisoning		0 deaths per 100,000 persons
Seychelles	Lagraulet (1975) [60]			Case report	Poisoning with fish toxin		
Seychelles	Myers et al. (2009) [61]	Seychelles Child Development Study		Prospective longitudinal study	Effects of methyl mercury exposure	779	Recent postnatal exposure at 107 months of age was adversely associated with four endpoints, but no consistent pattern
Uganda	WHO (2009) [4]	National statistics	2004	Burden of disease estimation	Mortality from unintentional poisoning		11.4 deaths per 100,000 persons
Uganda	Bwibo (1969) [62]	New Mulago Hospital	Jan 1963–Dec 1968	Retrospective case review	Accidental poisoning in children	130	Admission rate for accidental poisoning in children was 0.65% Case fatality rate was 5.4%; household chemicals accounted for largest number of poisonings
Uganda	Cardozo and Mugerwa (1972) [63]	Mulago Hospital, Kampala	Jan–Dec 1970	Retrospective hospital based case review	Acute poisoning	70	48 cases were children, accounting for 0.75% of total paediatric admissions for the period; most admissions were for kerosene ingestion 22 cases were adults, accounting for 0.35% of the total admissions; pesticide poisoning was the most common cause
Uganda	Kinyanda et al. (2004) [64]	Kampala	Nov 2001–Oct 2002	Case-control study	Deliberate self-harm (DSH)	100 cases of DSH; 300 controls	Poisoning was the most important method used in DSH (65%). Organophosphates accounted for the highest proportion (45%) with medications accounting for (35%)
Uganda	Malangu (2008) [65]	Two Hospitals in Kampala	Jan–June 2005	Retrospective hospital based case review	Acute poisoning	276	Agrochemicals (42.4%) were responsible for most of the admitted cases that presented for treatment, followed by household chemicals (22.1%), carbon monoxide (20%), snakebites (14.1%), and food poisoning (1.4%) The overall case fatality rate was 1.4%, due to alcohol, carbon monoxide, and organophosphates
Uganda	Office of the President (2009) [66]	Nationwide	2009	Press release	Methanol poisoning	27	19 deaths
Uganda	Digital Journal (2010) [67]	Southwest Uganda	2010	Media report	Methanol poisoning	189	89 deaths
United Republic of Tanzania	WHO (2009) [4]	National statistics	2004	Burden of disease estimation	Mortality from unintentional poisoning		6.6 deaths per 100,000 persons
United Republic of Tanzania	Rwiza (1991) [68]	Usangi Government Hospital	Jun 1981	Hospital based case series	*Datura stramonium* food contamination	10	No fatalities recorded
United Republic of Tanzania	Yates et al. (2010) [69]	Snake Park Clinic, Meserani	Apr 2007–Dec 2009	Clinic based prospective case series	Management of snakebites	85	42 cases received antivenom; the case fatality rate was 1% (1 death in a 12-year-old), while 7% had a skin graft or amputation of a limb or digit
United Republic of Tanzania	Mbakaya et al. 1994 [32]	Hospitals	1989/1990	Retrospective case review	Pesticide poisoning	736	736 cases of organochlorine poisoning during study period
United Republic of Tanzania	Howlett et al. (1990, 1992) [70, 71]	Tarime District	1985	Case review	Konzo associated with cassava consumption		118 cases including 2 verified deaths
United Republic of Tanzania	Mlingi et al. (1991) [72]	Msasi District	1988	Case review	Konzo associated with cassava consumption	3	
United Republic of Tanzania	Mlingi et al. (2011) [73]	Mbinga District Mtwara Region	2001/2002 & 2002/2003		Konzo associated with cassava consumption	24 cases (Mbinga) 214 cases (Mtwara)	
United Republic of Tanzania	Ndosi et al. (2004) [74]	Muhimbili Hospital, Dar Es Salaam		Prospective study of suicides	Poisoning	100 suicides	69% used poisoning, predominantly using antimalarials and pesticides
Zambia	WHO (2009) [4]	National statistics	2004	Burden of disease estimation	Mortality from unintentional poisoning		4.8 deaths per 100,000 persons
Zambia	Gill (1979) [75]	Hospitals in Chingola and Chililabombwe	Dec 1975–Jan 1978	Case series	Mushroom poisoning	14	The case fatality rate 14%
Zambia	Bhushan et al. (1979) [76]	University Teaching Hospital, Lusaka	1978	Retrospective hospital based case review	Accidental poisoning	378	Case fatality rate of 0.5% with paraffin poisoning accounting for the largest proportion of admissions (57.1%), food poisoning (18.3%), household poisons (11%), and medicines (10.8%)
Zambia	Gernaat et al. (1998) [77]	St. Paul's Hospital, Nchelenge	4 years	Combined retrospective and prospective study of admissions	Poisoning	6412	Main prevalence of snakebite was in ages of 4–14 yrs
Zambia	Sinclair et al. (1989) [78]	Hospital	16 months	Case series of nontraumatic coma	Poisoning	170	Organophosphate poisoning, a significant cause
Zimbabwe	WHO (2009) [4]	National statistics	2004	Burden of disease estimation	Mortality from unintentional poisoning		8 deaths per 100,000 people
Zimbabwe	Nhachi and Kasilo (1992) [79]	Six referral hospitals in Zimbabwe	1980–1989	Retrospective hospital case review	Admitted cases of poisoning	6018	Case fatality rate was 15%
Zimbabwe	Tagwireyi et al. (2002) [80]	Eight referral hospitals in Zimbabwe	Jan 1998–Dec 1999	Retrospective hospital case review	Admitted cases of poisoning	2764	Case fatality rate was 4.4% for all cases
Zimbabwe	Tagwireyi et al. (2006) [81]	Six district hospitals and one provincial hospital	Jan 1998–Dec 1999	Retrospective hospital case review	Admitted cases of poisoning	711 district hospital cases and 341 provincial cases	Case fatality rate was 4.8% (district hospitals) and 4.7% (provincial hospital)
Zimbabwe	Dong and Simon (2001) [82]	Parirenyatwa hospital	Jan 1995–Nov 2000	Retrospective hospital case review	Organophosphate poisoning	599	Most cases were due to deliberate self-poisoning (74%) The case fatality rate was 8.3% Admissions increased by 320% over the 6 years
Zimbabwe	Kasilo et al. (1991) [83]	Six referral hospitals	1980–1989	Retrospective hospital case review	Organophosphate poisoning	606	Most cases were due to deliberate self-poisoning (75%) The case fatality rate in the series was 8%
Zimbabwe	Nhachi (1988) [84]	One referral hospital and one district hospital	Jan 1981–Dec 1986	Retrospective hospital case review	Organophosphate poisoning	161 (urban); 11 (rural)	Most cases (83%) were intentional poisoning from urban and for rural centre most (70%) were accidental Case fatality rate 14% (urban)
Zimbabwe	Nyazema (1984) [85]	Two central hospitals and Government Analyst Laboratory	1971–1982	Retrospective case review	Number of cases of traditional medicine poisoning	?	297 cases admitted to Harare hospital for the period from 1971 to 1982
Zimbabwe	Kasilo and Nhachi (1992) [86, 87]	Six referral hospitals	1980–1989	Retrospective hospital case review	Traditional medicines poisoning	1456	Case fatality rate was 6%
Zimbabwe	Tagwireyi and Ball (2002) [88]	Parirenyatwa Central Hospital	Jan 1995–Dec 1999	Retrospective hospital case review	Traditional medicines poisoning in adults	16	No deaths reported
Zimbabwe	Tagwireyi et al. (2002) [89]	Eight referral hospitals in Zimbabwe	Jan 1998–Dec 1999	Retrospective hospital case review	Traditional medicines poisoning in adults	63	Case fatality rate was 9.5%
Zimbabwe	Tagwireyi and Ball (2002) [88]	Parirenyatwa Central Hospital	Jan 1995–Dec 1999	Retrospective hospital case review	Elephant's Ear poisoning	15	Clinical presentation and management of Elephant's Ear poisoning was described No deaths were reported
Zimbabwe	Flegg (1981) [90]		Mar 1980–Mar 1981	Retrospective hospital case review	Mushroom poisoning	50	Case fatality rate was 12%
Zimbabwe	Tagwireyi et al. (2002) [91]	Eight referral hospitals in Zimbabwe	Jan 1998–Dec 1999	Retrospective hospital case review	Acute poisoning in children (0–12 yrs)	761	97.5% admissions due to accidental poisoning Household chemicals especially paraffin responsible for largest proportion of admissions (43.2%) Case fatality rate was 3.1%
Zimbabwe	Chitsike (1994) [92]	Intensive care unit, Parirenyatwa Hospital	1990-1991	Retrospective hospital case review	Severe acute poisoning in children	42	Household chemicals especially paraffin responsible for largest proportion of admissions (26.2%) Case fatality rate of 21%
Zimbabwe	Kasilo and Nhachi (1992) [86, 87]	Six referral hospitals	1980–1989	Retrospective hospital case review	Acute poisoning in children (0–15 yrs)	2873	Most cases accidental (93.4%) Household chemicals especially paraffin responsible for largest proportion of admissions (27.2%) The case fatality rate of 4.9%
Zimbabwe	Blaylock (1982) [93]	Triangle District Hospital	Jan 1975–Jun 1981	Retrospective hospital case review	Snakebite	250	Case fatality rate of 0.4%
Zimbabwe	Geddes and Thomas (1985) [94]		?	Case report	Snakebite	1	Patient survived
Zimbabwe	Kasilo and Nhachi (1993) [95, 96]	Six referral hospitals	1980–1989	Retrospective hospital case review	Snakebite	995	Case fatality rate was 1.8%
Zimbabwe	Muguti et al. (1994) [97]	Mpilo Central Hospital	Jan 1990–Jun 1992	Retrospective hospital case review	Snakebite	83	Case fatality rate was 5%
Zimbabwe	Nhachi and Kasilo (1994) [98, 99]		Jan 1991–Dec 1992	Prospective	Snakebite poisoning	274	Case fatality rate was 1.8%
Zimbabwe	Muguti and Dube (1998) [100]	Mpilo Central Hospital	?	Case report	Snakebite from the vine snake *(Thelotornis capensis oatessi)*	1	Patient survived
Zimbabwe	Tagwireyi et al. (2004, 2011) [101, 102]	Eight referral hospitals	Jan 1998–Dec 1999	Retrospective hospital case review	Snakebite	273	Case fatality rate was 2.9%
Zimbabwe	Nhachi and Kasilo (1994) [98, 99]	Six referral hospitals	1980–1989	Retrospective hospital case review	Household chemical poisoning	1192	Majority of the cases (61.3%) occurred in the 0–5 years age group Case fatality rate of 13%
Zimbabwe	Tagwireyi et al. (2006) [103]	Eight referral hospitals	Jan 1998–Dec 1999	Retrospective hospital case review	Paraffin (Kerosene) poisoning	327	Most exposure instances (91.7%) occurred accidentally, with only 6.7% resulting from deliberate ingestion of the chemical The case fatality rate was 0.3%
Zimbabwe	Bergman (1997) [104, 105]	Rural clinics in Gwanda South District	Sep 1991–Sep 1993	Prospective hospital and clinic based survey	*Parabuthus transvaalicus* scorpionism		Case fatality rate was 0.3%; the mortality rate in the district was 2.8 per 100 000 per year
Zimbabwe	Saunders and Morar (1990) [106]			Case report	Scorpion sting	1	Patient survived without any specific scorpion antivenin administration
Zimbabwe	Nhachi and Kasilo (1993) [107]	Six referral hospitals	1980–1989	Retrospective hospital case review	Scorpion and insects poisoning	92	In scorpion sting/bite admissions, bees (44.6%), wasps (8.7%), and spiders (8,7%) accounted for most of the exposure instances No fatalities were recorded
Zimbabwe	Tagwireyi and Ball (2011) [108]	Eight referral hospitals	1998-1999	Retrospective hospital case review	Scorpion envenomation	29	No fatalities
Zimbabwe	Kasilo and Nhachi (1994) [109]	Six referral hospitals	1980–1989	Retrospective hospital case review	Food poisoning	487	Case fatality rate was 2.5%
Zimbabwe	Tagwireyi et al. (2000) [110]	A provincial hospital	1999	Case report	Cantharidin poisoning due to blister beetle ingestion	1	Patient survived
Zimbabwe	Nhachi et al. (1992) [111]	Six referral hospitals	1980–1989	Retrospective hospital case review	Therapeutic drugs poisoning	1061	Pharmaceutical poisoning admissions resulted from mainly accidental exposure (63.5%) The case fatality rate was 3.9%
Zimbabwe	Queen et al. (1999) [112]		May 1987–April 1995	Retrospective hospital case review	Chloroquine overdose	?	Preponderance of females taking chloroquine in overdose, compared to other overdoses and toxic exposure, was reported (OR 1.99; 95% CI 1.31–3.04; *p* < 0.001) Case fatality rate of 40%
Zimbabwe	McKenzie (1996) [113]		Nov 1990–Oct 1994	Retrospective hospital case review	Chloroquine overdose	29	Case fatality rate of 20.7%
Zimbabwe	Ball et al. (2002) [114]	Eight referral hospitals	Jan 1998–Dec 1999	Retrospective hospital case review	Chloroquine poisoning	544 (chloroquine 279)	Case fatality rate due to chloroquine poisoning significantly higher than that of poisoning due to other drugs (5.7% versus 0.7%; *p* < 0.0001)
Zimbabwe	Tagwireyi et al. (2006) [81]	Six referral hospitals and one provincial hospital	Jan 1998–Dec 1999	Retrospective hospital case review	Differences and similarities in poisoning admissions in urban and rural health centres	711 (district hospital); 341 (provincial hospital)	Case fatality rate for district hospitals was 4.8% Case fatality rate for provincial hospital was 4.7%
Zimbabwe	Tagwireyi et al. (2006) [115]	Eight referral hospitals	Jan 1998–Dec 1999	Retrospective hospital case review	Pesticide poisoning	914	Almost half (49.1%) resulted from oral exposure to rodenticides, 42.2% from anticholinesterase-type pesticides (AChTP) The case fatality rate was 6.8%
Zimbabwe	Kasilo and Nhachi (1993) [96]	Six referral hospitals	1980–1989	Retrospective hospital case review	Metal poisoning	40	Copper accounted for the largest proportion (27.5%)

## References

[1] Marks C., van Hoving N., Edwards N. (2016). A promising poison information centre model for Africa. *The African Journal of Emergency Medicine*.

[2] WHO (2015). *Improving the Availability of Poison Centre Services in Africa*.

[3] Bertrand P. G., Ahmed H. A. M., Ngwafor R., Frazzoli C. (2016). Toxicovigilance systems and practices in Africa. *Toxics*.

[4] WHO (2009). *Death and DALY Estimates for 2004 by Cause for Countries: Persons, All Ages by Country*.

[5] Benois A., Petitjeans F., Raynaud L., Dardare E., Sergent H. (2009). Clinical and therapeutic aspects of childhood kerosene poisoning in Djibouti. *Tropical Doctor*.

[6] Seignot P., Ducourau J. P., Ducrot P., Angel G., Roussel L., Aubert M. (1992). Fatal poisoning by an African viper's bite (*Echis carinatus*). *Annales Françaises d'Anesthésie et de Réanimation*.

[7] Larréché S., Mion G., Mayet A. (2011). Antivenin remains effective against African Viperidae bites despite a delayed treatment. *The American Journal of Emergency Medicine*.

[8] Aigle L., Lions C., Mottier F. (2010). Management of bluespotted stingray injuries in Djibouti from July 2008 to July 2009. *Médecine Tropicale*.

[9] Abebe M. (1991). Organophosphate pesticide poisoning in 50 Ethiopian patients. *Ethiopian Medical Journal*.

[10] Aseffa A., Mengistu G., Tiruneh M. (1994). Salmonella newport: outbreak of food poisoning among college students due to contaminated undercooked eggs. *Ethiopian Medical Journal*.

[11] Alem A., Kebede D., Jacobsson L., Kullgren G. (1999). Suicide attempts among adults in Butajira, Ethiopia. *Acta Psychiatrica Scandinavica*.

[12] Abula T., Wondmikun Y. (2006). The pattern of acute poisoning in a teaching hospital, north-west Ethiopia. *Ethiopian Medical Journal*.

[13] Melaku Z., Alemayehu M., Oli K., Tizazu G. (2006). Pattern of admissions to the medical intensive care unit of Addis Ababa University Teaching Hospital. *Ethiopian Medical Journal*.

[14] Desalew M., Aklilu A., Amanuel A., Addisu M., Ethiopia T. (2011). Pattern of acute adult poisoning at Tikur Anbessa specialized teaching hospital, a retrospective study, Ethiopia. *Human & Experimental Toxicology*.

[15] Azahz A. (2011). Severe organophosphate poisoning with delayed cholinergic crisis, intermediate syndrome and organophosphate induced delayed polyneuropathy on succession. *Ethiopian Journal of Health Science*.

[16] Selassie P. (1998). Organophosphate poisoning in Jimma. *Ethiopian Journal of Health Sciences*.

[17] Makita K., Desissa F., Teklu A., Zewde G., Grace D. (2012). Risk assessment of staphylococcal poisoning due to consumption of informally-marketed milk and home-made yoghurt in Debre Zeit, Ethiopia. *International Journal of Food Microbiology*.

[18] Aga A., Geyid A. (1992). An outbreak of acute toxicity caused by eating food contaminated with Datura stramonium. *Ethiopian Journal of Health Development*.

[19] Charters A. D. (1957). Mushroom poisoning in Kenya. *Transactions of the Royal Society of Tropical Medicine and Hygiene*.

[20] Davidson R. A. (1970). Case of African cobra bite. *The British Medical Journal*.

[21] Mwangemi P. M. (1976). Poisonous snake bite—a reappraisal. *East African Medical Journal*.

[22] Greenham R. (1978). Spitting Cobra (*Naja mossambica pallida*) bite in a Kenyan child. *Transactions of the Royal Society of Tropical Medicine and Hygiene*.

[23] Smith D. H., Timms G. L., Refai M. (1979). Outbreak of botulism in Kenyan nomads. *Annals of Tropical Medicine and Parasitology*.

[24] Kahuho S. K. (1980). Occasional report: drug poisoning in the intensive Intensive Care Unit, Kenyatta National Hospital. *East African Medical Journal*.

[25] Ngindu A., Kenya P. R., Ocheng D. M. (1982). Outbreak of acute hepatitis caused by aflatoxin poisoning in Kenya. *The Lancet*.

[26] Snow R. W., Bronzan R., Roques T., Nyamawi C., Murphy S., Marsh K. (1994). The prevalence and morbidity of snake bite and treatment-seeking behaviour among a rural Kenyan population. *Annals of Tropical Medicine and Parasitology*.

[27] Coombs M. D., Dunachie S. J., Brooker S., Haynes J., Church J., Warrell D. A. (1997). Snake bites in Kenya: a preliminary survey of four areas. *Transactions of the Royal Society of Tropical Medicine and Hygiene*.

[28] Centers for Disease Control and Prevention (CDC) (2004). Outbreak of aflatoxin poisoning—eastern and central provinces, Kenya, January–July 2004. *Morbidity and Mortality Weekly Report*.

[29] Maitai C. K., Kibwage I. O., Guantai A. N., Ombega J. N., Ndemo F. A. (1998). A retrospective study of childhood poisoning in Kenya in 1991– 93. *East and Central African Journal of Pharmaceutical Sciences*.

[30] Lang T., Thuo N., Akech S. (2008). Accidental paraffin poisoning in Kenyan children. *Tropical Medicine & International Health*.

[31] Musumba C. O., Pamba A. O., Sasi P. A., English M., Maitland K. (2004). Salicylate poisoning in children: report of three cases. *East African Medical Journal*.

[32] Mbakaya C., Ohayo-Mitoko G., Ngowi V. (1994). The status of pesticide usage in East Africa. *African Journal of Health Sciences*.

[33] BBC News http://news.bbc.co.uk/2/hi/africa/1032331.stm.

[34] Ministry of Environment and Mineral Resources Kenya (2011). *Kenya National Profile to Assess the Chemicals Management*.

[35] Nyamu D. G., Maitai C. K., Mecca L. W., Mwangangi E. M. (2012). Trends of acute poisoning cases occurring at the Kenyatta National Hospital, Nairobi, Kenya. *East and Central African Journal of Pharmaceutical Sciences*.

[36] Vicens R., Rasolofonirina N., Coulanges P. (1986). First human cases of food-induced botulism in Madagascar. *Archives de l'Institut Pasteur de Madagascar*.

[37] Domergue C. A. (1989). A venomous snake of Madagascar. 2 case reports of bites by Madagascarophis (Colubrida opisthoglypha). *Archives de l'Institut Pasteur de Madagascar*.

[38] Habermehl G. G., Krebs H. C., Rasoanaivo P., Ramialiharisoa A. (1994). Severe ciguatera poisoning in Madagascar: a case report. *Toxicon*.

[39] Ramialiharisoa A., De Haro L., Jouglard J., Goyffon M. (1994). Latrodectism in Madagascar. *Medecine Tropicale*.

[40] Ramialiharisoa A., Rafenoherimanana R., de Haro L., Jouglard J. (1996). Collective poisoning of ciguateric type after ingestion of shark in Madagascar. Data collected by the Antananarivo medical team. *Presse Médicale*.

[41] Ramialiharisoa A., Razafindraktoto L., Rasoanaivo P. (1997). Shark poisoning in Madagascar: a case report. *African Journal of Health Sciences*.

[42] Ranaivoson G., Champetier de Ribes G., Mamy E. R., Jeannerod G., Razafinjato P., Chanteau S. (1994). Mass food poisoning after eating sea turtle in the Antalaha district. *Archives de l"Institut Pasteur de Madagascar*.

[43] Boisier P., Ranaivoson G., Rasolofonirina N. (1994). Fatal ichthyosarcotoxism after eating shark meat. Implications of two new marine toxins. *Archives de l'Institut Pasteur de Madagascar*.

[44] Champetier De Ribes G., Ranaivoson G., Ravaonindrina N. (1998). Un problème de santé réémergent à Madagascar: les intoxications collectives par consommation d'animaux marins. Aspects épidémiologiques, cliniques et toxicologiques des épisodes notifiés de janvier 1993 à janvier 1998. *Archives de l'Institut Pasteur de Madagascar*.

[45] Ribes G. C., Ramarokoto S., Rabearintsoa S. (1999). Seafood poisoning in Madagascar: current state of knowledge and results of a retrospective study of the inhabitants of coastal villages. *Sante*.

[46] Robinson R., Champetier R. G., Ranaivoson G., Rejely M., Rabeson D. (1999). KAP study (knowledge-attitude-practice) on seafood poisoning on the southwest coast of Madagascar. *Bulletin de la Société de Pathologie Exotique*.

[47] Ravaonindrina N., Andriamaso T. H., Rasolofonirina N. (2001). Puffer fish poisoning in Madagascar: four case reports. *Archives de l'Institut Pasteur de Madagascar*.

[48] O'Reilly D. A., Heikens G. T. (2011). Organophosphate poisoning in a 12-day-old infant: case report. *Annals of Tropical Paediatrics*.

[49] Chibwana C., Mhango T., Molyneux E. M. (2001). Childhood poisoning at the Queen Elizabeth Central Hospital, Blantyre, Malawi. *East African Medical Journal*.

[50] Dzamalala C. P., Milner D. A., Liomba N. G. (2006). Suicide in Blantyre, Malawi (2000–2003). *Journal of Clinical Forensic Medicine*.

[51] Yu K. L., Bong C. N., Huang M. C. (2009). The use of hospital medical records for child injury surveillance in northern Malawi. *Tropical Doctor*.

[52] Glaizal M., Tichadou L., Drouet G., Hayek-Lanthois M., De Haro L. (2011). Ciguatera contracted by French tourists in Mauritius recurs in Senegal. *Clinical Toxicology*.

[53] Ministry Of Health-Mozambique (1984). Mantakassa: an epidemic of spastic paraparesis associated with chronic cyanide intoxication in a cassava staple area of Mozambique. 1. Epidemiology and clinical and laboratory findings in patients. Ministry of Health, Mozambique. *Bulletin of the World Health Organization*.

[54] Cliff J., Essers S., Rosling H. (1986). Ankle clonus correlating with cyanide intake from cassava in rural children from Mozambique. *Journal of Tropical Pediatrics*.

[55] Casadei E., Cliff J., Neves J. (1990). Surveillance of urinary thiocyanate concentration after epidemic spastic paraparesis in Mozambique. *Journal of Tropical Medicine and Hygiene*.

[56] Cliff J., Coutinho J. (1995). Acute intoxication from newly-introduced cassava during drought in Mozambique. *Tropical doctor*.

[57] Cliff J., Nicala D., Saute F. (1997). Konzo associated with war in Mozambique. *Tropical Medicine & International Health*.

[58] Cliff J., Nicala D., Saute F. (1999). Ankle clonus and thiocyanate, linamarin, and inorganic sulphate excretion in school children in communities with konzo, Mozambique. *Journal of Tropical Pediatrics*.

[59] Ernesto M., Cardoso A. P., Nicala D. (2002). Persistent konzo and cyanogen toxicity from cassava in northern Mozambique. *Acta Tropica*.

[60] Lagraulet J. (1975). Ichtyotoxism in the Seychelles Islands. *Bulletin de la Societé de Pathologie Exotique et de ses Filiales*.

[61] Myers G. J., Thurston S. W., Pearson A. T. (2009). Postnatal exposure to methyl mercury from fish consumption: a review and new data from the Seychelles Child Development Study. *NeuroToxicology*.

[62] Bwibo N. O. (1969). Accidental poisoning in children in Uganda. *British Medical Journal*.

[63] Cardozo L. J., Mugerwa R. D. (1972). The pattern of acute poisoning in Uganda. *East African Medical Journal*.

[64] Kinyanda E., Hjelmeland H., Musisi S. (2004). Deliberate self-harm as seen in Kampala, Uganda. *Social Psychiatry and Psychiatric Epidemiology*.

[65] Malangu N. (2008). Acute poisoning at two hospitals in Kampala-Uganda. *Journal of Forensic and Legal Medicine*.

[66] Uganda Office of the President 2009 Press release on methanol poisoning. http://www.digitaljournal.com/article/291234#ixzz2Dbx447JW.

[67] DIGITAL JOURNAL, Home-made brew laced with methanol kills 89 in Uganda, April 2010, http://digitaljournal.com/article/291234#ixzz2Dbx447JW

[68] Rwiza H. T. (1991). Jimson weed food poisoning. An epidemic at Usangi rural government hospital. *Tropical and Geographical Medicine*.

[69] Yates V. M., Lebas E., Orpiay R., Bale B. J. (2010). Management of snakebites by the staff of a rural clinic: the impact of providing free antivenom in a nurse-led clinic in Meserani, Tanzania. *Annals of Tropical Medicine and Parasitology*.

[70] Howlett W. P., Brubaker G. R., Mlingi N., Rosling H. (1990). Konzo, an epidemic upper motor neuron disease studied in Tanzania. *Brain*.

[71] Howlett W., Brubaker G., Mlingi N., Rosling H. (1992). A geographical cluster of konzo in Tanzania. *Journal of Tropical and Geographical Neurology*.

[72] Mlingi N., Kimatta S., Rosling H. (1991). Konzo, a paralytic disease observed in southern Tanzania. *Tropical Doctor*.

[73] Mlingi N. L. V., Nkya S., Tatala S. R., Rashid S., Bradbury J. H. (2011). Recurrence of konzo in southern Tanzania: rehabilitation and prevention using the wetting method. *Food and Chemical Toxicology*.

[74] Ndosi N. K., Mbonde M. P., Lyamuya E. (2004). Profile of suicide in Dar es Salaam. *East African Medical Journal*.

[75] Gill G. V. (1979). Mushroom poisoning in Zambia. *East African Medical Journal*.

[76] Bhushan V., Chintu C., Gupta K. (1979). Accidental poisoning in children in Lusaka. *Medical Journal of Zambia*.

[77] Gernaat H. B. P. E., Dechering W. H. J. C., Voorhoeve H. W. A. (1998). Clinical epidemiology of paediatric disease at Nchelenge, north-east Zambia. *Annals of Tropical Paediatrics*.

[78] Sinclair J. R., Watters D. A. K., Baghsaw A. (1989). Non-traumatic coma in Zambia. *Tropical Doctor*.

[79] Nhachi C. F. B., Kasilo O. M. J. (1992). The pattern of poisoning in urban Zimbabwe. *Journal of Applied Toxicology*.

[80] Tagwireyi D., Ball D. E., Nhachi C. F. B. (2002). Poisoning in Zimbabwe: a survey of eight major referral hospitals. *Journal of Applied Toxicology*.

[81] Tagwireyi D., Ball D. E., Nhachi C. F. B. (2006). Differences and similarities in poisoning admissions between urban and rural health centers in Zimbabwe. *Clinical Toxicology*.

[82] Dong X., Simon M. A. (2001). The epidemiology of organophosphate poisoning in urban Zimbabwe from 1995 to 2000. *International Journal of Occupational and Environmental Health*.

[83] Kasilo O. J., Hobane T., Nhachi C. F. B. (1991). Organophosphate poisoning in urban Zimbabwe. *Journal of Applied Toxicology*.

[84] Nhachi C. F. B. (1988). An evaluation of organophosphate poisoning cases in an urban setting in Zimbabwe. *East African Medical Journal*.

[85] Nyazema N. Z. (1984). Poisoning due to traditional remedies. *Central African Journal of Medicine*.

[86] Kasilo O. M. J., Nhachi C. F. B. (1992). A pattern of acute poisoning in children in urban Zimbabwe: ten years experience. *Human & Experimental Toxicology*.

[87] Kasilo O. M. J., Nhachi C. F. B. (1992). The pattern of poisoning from traditional medicines in urban Zimbabwe. *South African Medical Journal*.

[88] Tagwireyi D., Ball D. E. (2002). ‘Muti’ poisoning in Zimbabwe. *Tropical Doctor*.

[89] Tagwireyi D., Ball D. E., Nhachi C. F. B. (2002). Traditional medicine poisoning in Zimbabwe: clinical presentation and management in adults. *Human & Experimental Toxicology*.

[90] Flegg P. J. (1981). Mushroom poisoning. *The Central African Journal of Medicine*.

[91] Tagwireyi D., Ball D., Nhachi C. (2002). Childhood poisoning in Zimbabwe. *Journal of Toxicology. Clinical Toxicology*.

[92] Chitsike I. (1994). Acute poisoning in a paediatric intensive care unit in Harare. *The Central African Journal of Medicine*.

[93] Blaylock R. S. (1982). Snake bites at Triangle Hospital January 1975 to June 1981. *Central African Journal of Medicine*.

[94] Geddes J., Thomas J. E. P. (1985). Boomslang bite—a case report. *The Central African Journal of Medicine*.

[95] Kasilo O. M. J., Nhachi C. F. B. (1993). A retrospective study of poisoning due to snake venom in Zimbabwe. *Human & Experimental Toxicology*.

[96] Kasilo O. M. J., Nhachi C. F. B. (1993). Survey of chemical (mostly metals) poisoning cases as reflected in hospital admissions in urban Zimbabwe. *Bulletin of Environmental Contamination and Toxicology*.

[97] Muguti G. I., Maramba A., Washaya C. T. (1994). Snake bites in Zimbabwe: a clinical study with emphasis on the need for antivenom. *The Central African Journal of Medicine*.

[98] Nhachi C. F. B., Kasilo O. M. J. (1994). Household chemicals poisoning admissions in Zimbabwe's main urban centres. *Human & Experimental Toxicology*.

[99] Nhachi C. F. B., Kasilo O. M. J. (1994). Snake poisoning in rural Zimbabwe—a prospective study. *Journal of Applied Toxicology*.

[100] Muguti G. I., Dube M. (1998). Severe envenomation by a ‘pet’ vine snake. *The Central African Journal of Medicine*.

[101] Tagwireyi D., Ball D. E., Nhachi C. F. B. (2004). Snakebite admissions to major hospitals in zimbabwe. *Journal of Toxicology. Clinical Toxicology*.

[102] Tagwireyi D., Nhachi C. F., Ball D. E. (2011). Snakebite admissions in Zimbabwe: pattern, clinical presentation and management. *The Central African Journal of Medicine*.

[103] Tagwireyi D., Ball D. E., Nhachi C. F. B. (2006). Toxico-epidemiology in Zimbabwe: admissions resulting from exposure to paraffin (Kerosene). *Clinical Toxicology*.

[104] Bergman N. J. (1997). Scorpion sting in Zimbabwe. *South African Medical Journal*.

[105] Bergman N. J. (1997). Clinical description of *Parabuthus transvaalicus* scorpionism in Zimbabwe. *Toxicon*.

[106] Saunders C. R., Morar A. B. (1990). Beware the scorpion *Parabuthus*. *The Central African Journal of Medicine*.

[107] Nhachi C. F. B., Kasilo O. M. J. (1993). Poisoning due to insect and scorpion stings/bites. *Human & Experimental Toxicology*.

[108] Tagwireyi D., Ball D. E. (2011). Hospital admissions due to scorpion sting in zimbabwe. *Journal of Applied Sciences in Southern Africa*.

[109] Kasilo O. M. J., Nhachi C. F. B. (1994). Food poisoning admissions in referral hospitals in Zimbabwe: a retrospective study. *Human & Experimental Toxicology*.

[110] Tagwireyi D., Ball D. E., Loga P. J., Moyo S. (2000). Cantharidin poisoning due to ‘Blister Beetle’ ingestion. *Toxicon*.

[111] Nhachi C. F. B., Habane T., Satumba P., Kasilo O. M. J. (1992). Aspects of orthodox medicines (therapeutic drugs) poisoning in urban Zimbabwe. *Human & Experimental Toxicology*.

[112] Queen H. F., Tapfumaneyi C., Lewis R. J. (1999). The rising incidence of serious chloroquine overdose in Harare, Zimbabwe: emergency department surveillance in the developing world. *Tropical Doctor*.

[113] McKenzie A. G. (1996). Intensive therapy for chloroquine poisoning. A review of 29 cases. *South African Medical Journal*.

[114] Ball D. E., Tagwireyi D., Nhachi C. F. B. (2002). Chloroquine poisoning in Zimbabwe: a toxicoepidemiological study. *Journal of Applied Toxicology*.

[115] Tagwireyi D., Ball D. E., Nhachi C. F. B. (2006). Toxicoepidemiology in Zimbabwe: pesticide poisoning admissions to major hospitals. *Clinical Toxicology*.

[116] Akida A., Isinta M., Ndiawo F., Agedo D., Tsinanga J. (2011). (P2-54) legislation shaped by an emergency: methanol poisoning experience at Kenyatta National Hospital, Kenya. *Prehospital and Disaster Medicine*.

[117] The New Times Over 200 hospitalised after food poisoning. http://www.newtimes.co.rw/news/index.php?i=15008&a=54217.

[118] The Rwanda Express 50 Residents admitted over food poisoning. http://rwandaexpress.blogspot.com/2012/06/rwanda-50-residents-admitted-over-food.html.

[119] Lubwama S. W. (1985). Human *Salmonella serotypes* in Uganda, 1967–1982. *East African Medical Journal*.

[120] Nyazema N. Z. (1986). Herbal toxicity in Zimbabwe. *Transactions of the Royal Society of Tropical Medicine and Hygiene*.

[121] Tagwireyi D., Ball D. E. (2001). The management of Elephant's Ear poisoning. *Human & Experimental Toxicology*.

